# More than just one man and his dog: The many impacts of puppy acquisition on the mental health of families including children in the UK

**DOI:** 10.1371/journal.pone.0331179

**Published:** 2025-09-17

**Authors:** Zoe Belshaw, Claire L. Brand, Dan G. O’Neill, Rowena M. A. Packer

**Affiliations:** 1 EviVet Evidence-Based Veterinary Consultancy, Nottingham, United Kingdom; 2 Department of Clinical Science and Services, The Royal Veterinary College, Hatfield, Herts, United Kingdom; 3 Department of Pathobiology and Population Sciences, The Royal Veterinary College, Hatfield, Herts, United Kingdom; Federal University of Paraiba, BRAZIL

## Abstract

Many puppies were acquired during the COVID-19 pandemic to boost families’ mental health. The aim of this study was to characterise the nature, benefits and challenges of dog-child interactions as perceived by UK adult caregivers and co-habiting children aged 8–17 years. In 2023, a three-part online survey was administered incorporating qualitative and quantitative questions. We included two respondent groups: households owning (a) a puppy acquired during 2019 and (b) a puppy acquired during the COVID-19 pandemic in 2020–2021. Statistical analyses explored differences between ownership groups; free-text data was thematically analysed. Valid responses were collected from *n* = 382 caregivers and *n* = 216 children. Puppies’ primary adult caregivers were 95% female. Over one-third (37.3%) of caregivers found living with a puppy harder than expected; first-time owners were significantly more likely than experienced owners to find child-puppy interactions challenging. Almost all children were allowed to physically interact with their dog in ways previous studies have associated with an increased bite risk. Three themes were constructed from the free-text data, highlighting: (1) perceived positive aspects of dog ownership, particularly the importance to children and caregivers of close child-dog physical interactions; (2) challenges of managing a dog in a family home including negotiating responsibility for care between family members and establishing safe child-dog boundaries; (3) how one dog could differentially impact multiple household members, including that a single dog-person relationship could impact whole-household dynamics. These findings underscore the importance of involving all household members in human-dog research and highlight the unpredictability of whether acquisition motivations will align with outcomes. Resources are needed to help families safely and successfully integrate puppies into their homes, particularly in managing the evident associated maternal mental load.

## Introduction

Dog-owner relationships are typically researched as dyads of one dog-owner pair [1–4]. This tacitly assumes that each dog has only one adult owner and that all relevant information on that dog’s relationship with, and impact on, their human household can be derived from this person. However, it is estimated that approximately 1.6 million “family” homes in the United Kingdom (UK) contain adults, children, and at least one dog [5–9]. More than one person per household, including children, might therefore consider themselves the dog’s owner [10–13]. Dogs may be perceived to be integral family members in these homes [14,15], with children expected, and willing, to adopt varying levels of responsibility for their care depending on their age and what tasks their adult caregivers think are appropriate [12,16]. However, research is extremely limited on how co-habiting family members, including children, interact with and negotiate responsibility for the same dog. This paper seeks to start to fill that knowledge gap.

Dogs can be acquired by households for many reasons. Recently, mental health as a driver for acquisition has been highlighted; the COVID-19 pandemic led to a global surge in mental health problems amongst adults [17] and children [18], and a concomitant surge in ‘Pandemic Puppy’ acquisition [19,20], particularly amongst families [5]. Packer *et al*. [5] identified that 38% of UK adult owners acquired their ‘Pandemic Puppy’ to enhance their own or their family’s mental health. Mental health problems are estimated to cost the UK economy at least £117.9 billion annually [21], and dog ownership is therefore a very appealing potential solution. However, evidence to support the link between dog ownership and a mental health benefit during the COVID-19 pandemic is inconsistent for both children [22] and adults [11,23–32]. Furthermore, reviews of the evidence underpinning assertions about the benefits of pet ownership have identified only equivocal impacts on quality of life [33], happiness [34], loneliness [35], wellbeing [36], and in supporting people with formally diagnosed mental health conditions [37]. A variety of explanations for these apparently null findings have been proposed. These include that the studies did not account for other influences on wellbeing intrinsic to the owner [3,11] and that hedonic adaptation (the return of happiness to its previous baseline after a positive or negative event) may nullify initial benefits of pet acquisition over time [34]. Additionally, much of the existing research relies on quantitative measures which may not fully capture the complex nuances of pet-owner relationships [3,37], particularly regarding children [10,12,22,38,39].

Published studies have yet to explore the effects from ‘Pandemic Puppy’ acquisition versus existing dog ownership on child and adult mental health, or person-dog relationships, within the same household. Furthermore, outcomes from ‘Pandemic Puppy’ acquisitions explicitly aimed at providing companionship or improving mental health for children and their parents during the COVID-19 pandemic are not known. The aim of this study was to characterise the nature, benefits and challenges of dog-child interactions in households of UK adult caregivers and co-habiting children aged 8–17 years as reported by adults and children. We also sought to explore differences in mental health impact and human-dog bond during and after the COVID-19 pandemic.

## Methods

### Ethical approval

This study received ethical approval from the Royal Veterinary College Social Science Research Ethical Review Board (SSRERB), reference: SR2023−0010. Adult participants provided written informed consent to participate for themselves and their child(ren); children then provided assent for their own participation at the start of their survey section.

### Participant inclusion criteria and recruitment

A survey was designed for completion by UK-resident adults and children. Eligible adult participants had to meet the following three inclusion criteria:

a) Having caregiving responsibility for at least one co-habiting child aged 8–17 years inclusive at the time of survey completion.
b) Ongoing ownership of a dog purchased when aged under 16 weeks (of any breed or a crossbreed) from a private seller (i.e., anyone selling a puppy but not a shelter) between 23rd March – 31st December 2019 (a ‘2019 puppy’), or within the same date-range in 2020 or 2021 (a ‘Pandemic Puppy’).
c) Being the primary caregiver for that dog.

A private seller was specified rather just than a breeder (defined here as the person who bred the puppy) due to the number of illegal sellers operating during the COVID-19 pandemic; the new purchaser might have assumed the seller to be a breeder, but this may not have been the case. Shelter adoptions were excluded as we were interested in comparing puppies bought direct from sellers. The inclusion start date of 23rd March reflects the start of the first COVID-19 lockdown in the UK.

Any number of children aged 8–17 years resident within each household were also eligible to participate if a co-habiting adult caregiver had completed the adult portion of the survey. Only one combined caregiver-child(ren) response was permitted per household.

The majority of respondents were recruited from the original 'Pandemic Puppies' study cohort [5,40], hereafter termed ‘Recruitment Group A’. That survey-based research compared a large cohort of UK ‘Pandemic Puppies’ acquired from private sellers in 2020–2021 with puppies acquired via the same route in 2019 to determine whether ‘Pandemic Puppies’ had any unique welfare risks or ongoing problems specific to the timing and manner of their acquisition. Recruitment Group A respondents who fulfilled our inclusion criteria were contacted if they had indicated willingness to participate in further research and provided a valid email address. These participants (*n* = 1258) were sent a unique questionnaire link on 28th February 2023; this ensured only one response could be collected per household. Reminder emails were sent automatically every two weeks during the data collection period if potential participants had not completed the survey. This survey version closed on 9th May 2023.

A second version of the survey was disseminated via snowball sampling across social media using a virtual flyer shared by various associates and groups (see acknowledgements for full list) to recruit any other participants who met the inclusion criteria. This version of the survey was open from 24th March – 14th May 2023, and its respondents are hereafter termed ‘Recruitment Group B’. To both groups, the study was advertised as “a survey for adults and children to explore whether dogs supported children’s mental health during the pandemic”. Respondents from both recruitment groups were pooled, then re-grouped based on when their dog was acquired into a) the ‘2019 puppy group’ for dogs acquired in 2019, or b) the ‘Pandemic Puppy group’ for dogs acquired in 2020 and 2021.

### Survey design

The authors iteratively designed surveys for Recruitment Groups A and B (see S1 Appendix). Both were piloted by a small number of eligible caregivers and children before launch. The surveys each consisted of three linked questionnaires, the first two to be completed by the caregiver and the third by any or all their eligible children. Only the initial inclusion criteria questions were mandatory. The survey versions differed in two ways: a) caregiver respondents from Recruitment Group A were asked to confirm continued ownership of their dog before proceeding with the first questionnaire; and b) caregiver respondents in Recruitment Group B were asked questions about their dog’s purchase that had previously been presented to Recruitment Group A respondents in the original 2020 survey [5,40]. Both surveys were hosted on Vanderbilt University’s Research Electronic Data Capture platform (REDCap), part of the REDCap Consortium [41,42]. Families with more than one eligible dog were asked to focus on their relationship with the youngest dog and its impacts. If dogs were of the same age, respondents were asked to consider the dog whose name came first alphabetically.

The surveys were designed in three parts as detailed below. The first two parts were designed to be completed by the caregiver, focussed on their own relationship with the dog and their own mental health (Caregiver Questionnaire 1), and their perspectives on their child(ren)’s relationship with the dog and their child(ren)'s mental health (Caregiver Questionnaire 2). The third part, to be answered by any or all of their eligible children, comprised a child’s reflection on their own mental health and their relationship with the dog (Child Questionnaire). The surveys contained quantitative measures to assess caregiver and child(ren)’s mental health and bond with their dog. Due to the low number of respondents in the 2019 ownership group, the results from these measures and other statistical comparisons between acquisition groups were underpowered and therefore no strong conclusions can be drawn. For completeness, the related methods and results are presented in S2 Appendix but they do not feature further in the main body of the paper.

1. Caregiver Questionnaire 1:
  (i) *Initial questions*: Recruitment Group A were asked whether they still owned their dog. Those who did not were invited to optionally provide details about why not before exiting the survey. Recruitment Group B were asked to provide data on prior dog ownership experience, pre-purchase and purchase motivations for acquiring their puppy.
  (ii) *Demographic questions*: Recruitment Group A updated demographic variables that could have changed over time. Caregiver, household and dog demographics were collected from Recruitment Group B. After this point, the surveys were identical.
  (iii) *Perceptions and management of puppy/dog behaviour*: Caregivers completed multiple choice and/or free-text questions about:
    - expectations versus realities of puppy ownership: toilet training; managing biting/chewing of household objects; managing nipping/biting of children during play; and managing interactions between their child(ren) and the puppy (adapted from Packer *et al*. [43]).
    - any current concerns regarding their dog’s interactions with their own and visiting children.
    - whether they permitted any of 25 specified child-dog activities or interactions (e.g., “Hug your dog”; “Pick your dog up”; “Tell your dog off verbally” (adapted from Arhant *et al*. [44]).
    - whether they had considered rehoming their dog at any stage.
2. Caregiver Questionnaire 2:
  (i) *Demographic questions*: Caregivers provided demographic details of each eligible child in the household.
  (ii) *Perceptions of child involvement in dog care/child-dog bond*: Caregivers reported their expectations versus realities of each child’s involvement in feeding, walking, grooming, taking responsibility for the puppy/dog, and playing with the puppy/dog, adapted from Packer *et al*. [43]. This comparison was made at two timepoints: until the puppy reached 6 months of age; and at the time of survey completion. Finally, caregivers were asked their perception of each child’s relationship with the dog at the time of survey completion versus when the dog was acquired.
3. Child Questionnaire: This questionnaire was completed by each eligible child who agreed to participate once their caregiver had completed their questionnaires. The children completed:
  (i) *Demographic questions*: Their age and gender.
  (ii) *Child-dog interactions*: Three multiple choice and free-text questions: time currently spent with their dog; their current involvement in their dog’s care; and descriptions of how they recalled that their dog had made life better or worse during the COVID-19 lockdowns.

### Quantitative data analysis

Raw quantitative data were exported from REDCap into Microsoft Excel for Mac, v16.86 for manual cleaning prior to analysis. Responses from ineligible respondents and those whose survey was blank after the consent questions were removed. If respondents had not completed in full any of the question sets relating to permitted child-dog activities and interactions or expectations versus realities of puppy ownership, their responses were excluded from that analysis. Incomplete responses were otherwise included, including eligible caregiver responses with no corresponding child responses. Responses from Respondent Group B were manually checked for duplicates using dog name and acquisition month. IBM SPSS Statistics v29 (SPSS Inc., Chicago, IL., USA) was used to calculate descriptive statistics. Data distribution was assessed visually using histograms and the distribution of residuals. Non-normally distributed continuous data (child age in whole years) is reported as median (IQR, range). Chi-squared (*X*^*2*^) univariable analysis of caregiver expectations versus realities of puppy training/management and child involvement in dog care at the time of the survey by dog ownership experience included Bonferroni-corrections and post-hoc comparisons. Statistical power of tests were assessed using the Power Analysis option in SPSS. Statistical significance for all quantitative analyses was set at *p* < 0.05.

### Qualitative data analysis

Free-text responses were primarily analysed by ZB, with input from co-authors RMAP and CLB. Analysis employed a semantic, experiential, realist reflexive thematic analytic approach [45,46], which aims to capture explicit truths and realities from the dataset. Reporting is according to the relevant aspects of the COREQ checklist [47]. ZB’s analysis of these data was informed by her professional and dog-owning experiences. She is a female small animal veterinary surgeon with a PhD, and ongoing research interest in, pet-owner interactions. She is also a previous dog owner, and mother to a young child who is nervous around dogs. The other co-authors involved in qualitative analyses (RMAP, CLB) are experienced canine researchers, parents and dog owners. Our experiences and expertise are acknowledged to have informed, and introduced some subjectivity to, the analysis of these data.

Initial analysis of the free-text data revealed no substantial differences between caregivers or children who acquired puppies in 2019 and those who bought 'Pandemic Puppies'. Therefore, qualitative data from 2019 and 'Pandemic Puppy' groups were analysed together in a multi-step process. Initially, responses to each free-text-only question in both caregiver and child questionnaires were inductively coded into multiple small semantic codes. This was done using pen-and-paper then Microsoft Excel (Microsoft Windows 2016). Codes were then collated across the whole dataset. Caregiver and child responses for each household were also collated to create a more cohesive understanding of the roles and perceptions of each dog within individual households. Codes were serially grouped and re-grouped, then developed into candidate themes using paper-based thematic mapping [48]. Finally, further refinement led to the three inductive themes presented below. Due to the nature of data collection, participants were not able to provide feedback on the analysis.

Pseudonymised illustrative quotes are included below, with information provided on the participant’s group and basic demographic data. Inclusion of […] in a quote denotes words have been removed for brevity. The term “dog” has been used to encompass puppies and dogs of any age other than where the responses are specific to puppies.

The quantitative results are presented followed by the qualitative ones for ease of reading; comparisons between the two datasets are made in the discussion.

## Results

### Demographic data

**Fig 1:**
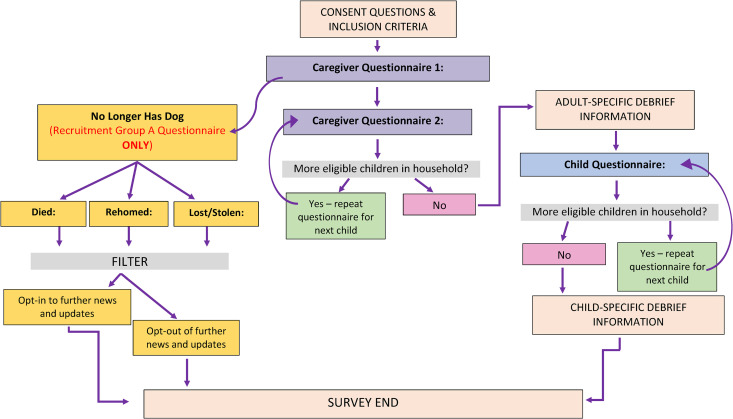
Schematic representation of the individual questionnaire responses and manual cleaning process prior to full analysis of the survey data. Households did not have to complete all three linked questionnaires within the overall survey to be included in analyses.

Four hundred and sixty-four responses to the survey were received (*n* = 376 from Respondent Group A and *n* = 88 responses from Respondent Group B). After data cleaning, *n* = 382 caregivers (82.3%; 2019 puppies group, *n* = 58; 'Pandemic Puppies' group, *n* = 324) remained who had provided a valid response about their households to Caregiver Questionnaire 1. Respondents from *n =* 299 (78.3%) of those 382 households (2019 puppies group, *n* = 42 households; 'Pandemic Puppies' group, *n* = 257 households) provided valid responses to Caregiver Questionnaire 2, describing *n* = 362 children (2019 puppies group, *n* = 51; 'Pandemic Puppies' group, *n* = 311). Child Questionnaire responses were provided by *n* = 216 (60%) of those children (2019 puppies group, *n* = 34; 'Pandemic Puppies' group, *n* = 182), representing *n* = 185 separate households (2019 puppies group, *n* = 28 households; 'Pandemic Puppies' group, *n* = 158 households). Fig 1 details the responses to each of the individual questionnaires comprising the survey and the manual cleaning process. Given the low response rate in the 2019 puppies group, any statistical comparisons were underpowered, and the following quantitative results therefore refer to both groups as a whole.

### Caregiver and child demographics

The majority of caregivers were female (95.5%, *n* = 358/375), with just under half aged 45–54 years (47.6%, *n* = 179/376). In terms of dog ownership experience, 55.2% (*n* = 207/375) caregivers reported having previously owned a dog. Just over two-thirds of caregivers indicated they had undertaken pre-purchase research on dog ownership (67.5%, *n* = 257/381). Over half of caregivers had acquired their dog to improve their families’ mental health (53.7%, *n* = .205/382).

Among the *n* = 216 child respondents, 53.2% reported they were female (*n* = 115/216). Median child age was 13.00 (IQR = 10.00–15.00, range = 8.00 –– 17.00). Caregivers of 24.3% (*n* = 84/345) children reported seeking a diagnosis or support for their child’s mental health. Of these *n* = 84 caregivers 82.1% (*n* = 69/84) cited the COVID-19 pandemic as a contributing factor in those children needing mental health support.

### Child-dog interactions allowed by caregivers

All *n* = 301 caregivers who responded to the question about permitted child-dog interactions (Table 1) allowed their child(ren) to stroke or pat their dog, with the majority allowing their child(ren) to demonstrate other means of physical affection (hugging 95.0%, *n* = 286; kissing 89.0%, *n* = 268). Over one-third of parents (34.2%, *n* = 103,) allowed their child(ren) to interact with their dog whilst their dog was eating, with a minority (6.6%, *n* = 20) allowing their child(ren) to pull on their dog’s ears or tail. Seventeen of the 25 listed interactions (highlighted * in Table 1) have previously been reported to be associated with an increased risk of dog bites [38,49]; over 50.0% of children were permitted to perform eight of these.

**Table 1:** Permitted child-dog interactions collated from caregivers in response to the multiple-choice question “Which of the following is your child/children allowed to do with your dog?” (*n* = 301).

Permitted child-dog interaction	*n*	%
Stroke or pat your dog on its body	301	100.0
Give your dog commands (e.g., sit, stay, etc.)	299	99.3
Approach or follow your dog*	289	96.0
Hug your dog*	286	95.0
Hold your dog’s lead whilst on walks	286	95.0
Stroke or pat your dog on its head NOT whilst it is eating or drinking	276	91.7
Groom your dog	270	89.7
Kiss your dog*	268	89.0
Lie down near your dog when it is resting*	259	86.0
Feed your dog their meals	255	84.7
Tell your dog off verbally	244	81.1
Take child toys/possessions away from your dog*	221	73.4
Yell, scream or be noisy whilst playing games with or around your dog*	197	65.4
Take away your dog’s toys*	187	62.1
Restrain your dog by its collar (directly, without a lead)*	165	54.8
Pick your dog up*	147	48.8
Involve your dog in their own games, e.g., doctors, vets, etc.*	138	45.8
Take away your dog’s food (including their bowl and/or dog chews) *	128	42.5
Wake your dog when it is sleeping*	104	34.6
Stroke or pat your dog when it is eating or drinking*	103	34.2
Physically correct your dog, e.g., tap your dog’s nose, etc.*	57	18.9
Dress your dog up in human/dolls clothes*	35	11.6
Throw objects at your dog*	29	9.6
Pull on your dog’s body parts, e.g., tail, ears*	20	6.6
Sit, lie or ride on your dog*	17	5.6

*Interactions with potential for increased bite risk, based on previous literature [38,49].

### Caregiver expectations versus realities of puppy/dog ownership

Just over one-third (37.3%, *n* = 139/373) of caregiver respondents to the questions regarding expectations versus realities of puppy training/management perceived at least one aspect of puppy training/management to have been worse than expected soon after acquisition. Of these, 30.2%, (*n* = 42/139) perceived two or more aspects to have been worse than expected. Dealing with their puppy nipping/biting their child during play was the aspect of puppy training/management perceived to be worse than expected by the most caregivers (Table 2).

**Table 2:** Caregiver expectations versus realities of puppy training/management soon after acquisition in response to the multiple-choice question “Thinking back to when your dog was a puppy, how did the following aspects of their care meet the expectations you had prior to acquiring them?”.

Expectation for puppy training/management by caregivers (total *n*)	Better/ easier than expected		As expected		Worse than expected		Not sure/ can’t remember	
	*n*	%	*n*	%	*n*	%	*n*	%
Toilet training (*n* = 371)	157	42.3	167	45.0	46	12.4	1	0.3
Dealing with biting/chewing of household objects (*n* = 372)	165	44.4	153	41.1	53	14.2	1	0.3
Dealing with nipping/biting children during play (*n* = 369)	136	36.9	168	45.5	65	17.6	0	0.0
Managing interactions between child and puppy (*n* = 370)	133	35.9	204	55.1	33	8.9	0	0.0

First-time dog owners were significantly more likely to have perceived dealing with their puppy nipping/biting their children as worse than expected compared to caregivers with previous ownership experience (first-time owner: 24.4% versus previous dog owner: 12.4%; *X*^*2*^ = 10.91, *p* = 0.004). First-time owners also found managing interactions between their child and a puppy worse than expected compared to those with previous ownership experience (first-time owner: 14.6% versus previous dog owner: 4.5%; *X*^*2*^ = 11.44, *p* = 0.003) (Table 3).

**Table 3:** Impact of previous dog ownership experience on caregiver expectations versus realities of puppy training/management soon after acquisition. Significant differences at the 5% level identified by post-hoc comparisons are highlighted by emboldened text.

Expectation for puppy care by caregivers	Expectation versus reality	Ownership status				Statistics	
		First- time dog owner		Previous ownership experience		*X* ^*2*^	*p*-Value
		*n*	%	*n*	%		
**Toilet training** (Total *n* = 367; 1^st^ time owner *n* = 166; previous ownership experience *n* = 201)	Better/easier than expected	78	47.0 ^a^	77	38.3 ^a^	5.14	0.162
	As expected	65	39.2 ^a^	100	49.8 ^a^		
	Worse than expected	23	13.9 ^a^	23	11.4 ^a^		
	Not sure/can’t remember	0	0.0	1	0.5 ^a^		
**Dealing with biting/chewing of household objects** (Total *n* = 368; 1^st^ time owner *n* = 166; previous ownership experience *n* = 202)	Better/easier than expected	69	41.6 ^a^	95	47.0 ^a^	6.51	0.089
	As expected	65	39.2 ^a^	86	42.6 ^a^		
	Worse than expected	31	18.7 ^a^	21	10.4 ^a^		
	Not sure/can’t remember	1	0.6 ^a^	0	0.0		
**Dealing with nipping/biting children during play** (Total *n* = 365; 1^st^ time owner *n* = 164; previous ownership experience *n* = 201)	**Better/easier than expected**	**50**	**30.5** ^**a**^	**86**	**42.8** ^**b**^	**10.91**	**0.004**
	As expected	74	45.1 ^a^	90	44.8 ^a^		
	**Worse than expected**	**40**	**24.4** ^**a**^	**25**	**12.4** ^**b**^		
	Not sure/can’t remember	0	0.0	0	0.0		
**Managing interactions between child and puppy** (Total *n* = 366; 1^st^ time owner *n* = 164; previous ownership experience *n* = 202)	Better/easier than expected	55	33.5 ^a^	77	38.1 ^a^	**11.44**	**0.003**
	As expected	85	51.8 ^a^	116	57.4 ^a^		
	**Worse than expected**	**24**	**14.6** ^**a**^	**9**	**4.5** ^**b**^		
	Not sure/can’t remember	0	0.0	0	0.0		

Significant differences at the 5% level identified by post-hoc comparisons are denoted by differing subscript letters (e.g., a, b) rather than identical subscript letters (e.g., a, a). For this analysis, Bonferroni-corrections and post-hoc comparisons were applied.

### Caregivers’ expectations versus realities of their children’s involvement in dog care

When considering their child(ren)’s change in involvement with aspects of their dog’s care at the time of survey completion compared to when their puppy was < 6 months of age, 49.2% of caregivers (*n* = 161/327) reported at least one aspect where their child(ren) were less involved than anticipated (Table 4). The aspects of dog care with the greatest increase over time in caregivers who felt their children were doing less than expected by the time of survey completion were walking (+11.9% increase) and feeding the dog (+9.6% increase). However, caregivers typically felt their children were currently more involved than expected, or as involved in as expected, in all five aspects of their dogs’ care. Dog walking was the aspect of care where the most caregivers felt involvement was greater than expected, whilst caregivers were most likely to score their children as currently less involved than expected in taking responsibility for the dog and playing with the dog.

**Table 4:** Expectations versus reality for child involvement in dog care by caregivers whilst their dog was < 6 months old (*n* = 335) and at the time of the survey in 2023 (*n* = 327).

Area of expectation	Expectation versus reality	Dog aged < 6 months (*n* = 335)		At time of the survey (*n* = 327)		Change over time (%)
		*n*	%	*n*	%	
**Feeding**	More than expected	40	11.9	36	11.0	−0.9
	As expected	244	72.8	212	64.8	−8.0
	Less than expected	48	14.3	78	23.9	+9.6
	Not sure/can’t remember	3	0.9	1	0.3	−0.6
**Walking**	More than expected	43	12.8	40	12.2	−0.6
	As expected	210	62.7	169	51.7	−11.0
	Less than expected	81	24.2	118	36.1	+11.9
	Not sure/can’t remember	1	0.3	0	0.0	−0.3
**Grooming**	More than expected	36	10.7	24	7.3	−3.4
	As expected	228	68.1	208	63.6	−4.5
	Less than expected	64	19.1	88	26.9	+7.8
	Not sure/can’t remember	7	2.1	7	2.1	0.0
**Taking responsibility**	More than expected	58	17.3	56	17.1	−0.2
	As expected	215	64.2	201	61.5	−2.7
	Less than expected	61	18.2	69	21.1	+2.9
	Not sure/can’t remember	1	0.3	1	0.3	0.0
**Playing**	More than expected	104	31.0	88	26.9	−4.1
	As expected	184	54.9	179	54.7	−0.2
	Less than expected	47	14.0	60	18.3	+4.3
	Not sure/can’t remember	0	0.0	0	0.0	0.0

Caregivers’ prior dog ownership experience was significantly associated with their expectations versus realities of child involvement in dog care (Table 5). First-time dog owners were significantly more likely to have rated their child’s current involvement in dog care as less than expected for all areas except feeding.

**Table 5:** Impact of previous dog ownership experience on caregiver expectations versus realities of child involvement in dog care at the time of the survey. Significant differences at the 5% level identified by post-hoc comparisons are highlighted by emboldened text.

Area of expectation	Expectation versus reality category	Ownership status				Statistics	
		First- time dog owner		Previous experience as a dog owner		*X* ^*2*^	*p*-Value
		n	%	n	%		
**Feeding** (Total *n* = 338; 1^st^ time owner *n* = 151; previous experience as a dog owner *n* = 187)	More than expected	14	9.3 ^a^	23	12.3 ^a^	6.682	0.083
	As expected	91	60.3 ^a^	128	68.4 ^a^		
	Less than expected	45	29.8 ^a^	36	19.3 ^a^		
	Not sure/can’t remember	1	0.7 ^a^	0	0.0		
**Walking** (Total *n* = 337; 1^st^ time owner *n* = 150; previous experience as a dog owner *n* = 187)	More than expected	15	10.0 ^a^	25	13.4 ^a^	**8.331**	**0.016**
	**As expected**	**69**	**46.0** ^**a**^	**108**	**57.8** ^**b**^		
	**Less than expected**	**66**	**44.0** ^**a**^	**54**	**28.9** ^**b**^		
	Not sure/can’t remember	0	0.0	0	0.0		
**Grooming** (Total *n* = 332; 1^st^ time owner *n* = 151; previous experience as a dog owner *n* = 181)	More than expected	10	6.6 ^a^	15	8.3 ^a^	**13.329**	**0.004**
	**As expected**	**84**	**55.6** ^**a**^	**128**	**70.7** ^**b**^		
	**Less than expected**	**51**	**33.8** ^**a**^	**37**	**20.4** ^**b**^		
	**Not sure/can’t remember**	**6**	**4.0** ^**a**^	**1**	**0.6** ^**b**^		
**Taking responsibility** (Total *n* = 335; 1^st^ time owner *n* = 150; previous experience as a dog owner *n* = 185)	**More than expected**	**18**	**12.0** ^**a**^	**38**	**20.5** ^**b**^	**16.923**	**< 0.001**
	As expected	87	58.0 ^a^	122	65.9 ^a^		
	**Less than expected**	**45**	**30.0** ^**a**^	**24**	**13.0** ^**b**^		
	Not sure/can’t remember	0	0.0	1	0.5		
**Playing** (Total *n* = 336; 1^st^ time owner *n* = 151; previous experience as a dog owner *n* = 185)	More than expected	34	22.5 ^a^	55	29.7 ^a^	**11.316**	**0.003**
	As expected	79	52.3 ^a^	109	58.9 ^a^		
	**Less than expected**	**38**	**25.2** ^**a**^	**21**	**11.4** ^**b**^		
	Not sure/can’t remember	0	0.0	0	0.0		

Significant differences at the 5% level identified by post-hoc comparisons are denoted by differing subscript letters (e.g., a, b) rather than identical subscript letters (e.g., a, a). For this analysis, Bonferroni-corrections and post-hoc comparisons were applied.

### Consideration of relinquishment

A total of 6.1% (*n* = 21/347) caregivers had considered (*n* = 19), or were considering (*n* = 2), rehoming their dog. Of these 21 caregivers, 85.7% (*n* = 18/21) indicated they were first-time dog owners. Free-text reasons provided for considered/considering relinquishment (*n* = 17) suggested two underlying causes. Either the caregiver had underestimated the time a puppy would need and/or struggled to manage their dog alongside their other responsibilities (*n* = 9/17; 52.9%) or the dog had behaviour problems (aggression, separation related behaviours or reactivity; *n* = 8/17; 47.1%), sometimes attributed to socialisation deficits during the pandemic. Five caregivers reflected that they wished they had known more about dogs, or their specific breed, before acquisition. For fourteen caregivers (82.4%), the situation had improved substantially as their dog matured as their lifestyle had largely normalised post-COVID-19 lockdowns and/or as a result of additional help with dog care and/or training. For the remaining three (*n* = 3/17; 17.6%), substantial behaviour problems continued.

### Qualitative data

Three themes were inductively constructed from qualitative data provided by all adult caregivers (*n* = 382) and *n* = 138/216 (63.9%) children, the latter from *n* = 122 households. The first theme, “The unconditional friend” focuses on the benefits family members identified from living with a dog. The theme name is taken from a caregiver’s description of their dog. The second theme “Owning a dog is harder than expected” focuses on some of the unanticipated challenges identified by families. It has two subthemes, “Whose responsibility is this?” and “Keeping everyone safe”, illustrating two aspects of these challenges. The final theme, “One dog, many impacts” focuses on the importance of looking beyond a single dog-owner dyad when considering the impact of a dog on the overall family.

#### Theme 1: The unconditional friend.

This first theme explores the multifaceted benefits and joys of owning a dog, both during the COVID-19 pandemic and beyond.

Almost all families reported that at least one family member had experienced enduring mental health benefits from sharing life with their dog. Many caregivers expressed amazement at the emotional closeness between their dog and child(ren), and the love their child(ren) felt for their dog. Children detailed enjoying various activities with their dogs including teaching them tricks, taking them for walks, showing them off to others, confiding in them, caring for them, and just being with them. They described their dogs as making made them feel happy, comforted, safe, energised and less lonely. Dog-child relationships were often described by both caregivers and children in human terms; dogs were termed a child’s friend, playmate, surrogate sibling, or even littermate.

*Someone to play with. Something to pet. Someone to confide in. Something to comfort me.* [11yo girl, household 32]

*My dog was always there when I was bored and she was someone I could talk to when parents are busy.* [11yo boy, household 64]

When not using their dog’s name, children often referred to “*my* dog”, whilst caregivers broadly discussed “*the* dog” or “*our* dog”. This difference is exemplified by the caregiver of the boy quoted above.

*My eldest child is reluctant to feed the dog due to the smell of the dog food…* [Female caregiver of two children, household 64]

Many caregivers reflected that the COVID-19 lockdowns had been very difficult. Children and adults in some family units had struggled with mental health, isolation, loneliness, and occasionally bereavement. The mental health of a few children, particularly those identified by their caregivers as neurodivergent, improved because of being off school during lockdowns. Others had hated the isolation and developed new, sometimes substantial, mental health problems. For many caregivers, the dog’s presence was recalled as being instrumental in providing a joyful, grounding focus during the COVID-19 lockdowns.

*For a long time [my younger son] would say that the dog was the only good thing in his life/only good thing that happened in lockdown (he really was very miserable and the dog was critically important to him).* [Female caregiver of two children, household 1]

*My children talk to our dog about their worries and sadness which was hugely important during the pandemic, especially as we suffered a huge bereavement.* [Female caregiver of three children, household 131]

Children echoed this, with most focusing on how their dog had made them feel less lonely and provided comfort during the lockdowns, helped them to get outside, or joined them in online lessons. Some recalled how the puppy/dog had helped them with specific challenges or had boosted the mental health of other family members.

*Had someone to be with when locked inside so didn’t feel as lonely I didn’t get to talk to my friends much apart from FaceTime.* [11yo boy, household 77]

*It was nice to have a companion and something to care for. It was nice to have a distraction, and my Mum was less fixated on COVID because of the dog.* [16yo girl, household 227]

Close physical contact appeared central to many children’s relationships with their dog. Most caregivers and children described children deriving great pleasure from cuddling, snuggling, kissing, hugging, and/or bed sharing with their dog. The close physical contact between children and their dogs seemed to have twin drivers: children’s demonstration of their love for their dogs; and the emotional boost provided by physical contact, especially during negative emotional states such as loneliness, anxiety, or sadness. Descriptions from both children and caregivers implied that dogs were perceived as willing participants in these physical interactions. Many caregivers expressed confidence that their dogs enjoyed these interactions as much as their children did. This was perhaps the reason that notably few caregivers mentioned potential risks associated with these behaviours, or the need for adult supervision.

*[Dog's name] is the unconditional friend. Always excited to see her and play. Always ready for a cuddle. Always engaged. [Dog's name] is the port in a storm. His fur mops up tears and his tongue licks away self-doubt*. [Female caregiver of one child, household 14]

Children spontaneously disclosed to be neurodivergent and those with no siblings were often identified by caregivers, and themselves, as having particularly special bonds with their dog. Close physical contact was again central to these relationships.

*My son [with neurodivergences] finds the dogs calming and loves them lying on him – it’s like a weighted blanket. He always cuddles them when feeling stressed this helps them to calm him.* [Female caregiver of one child, household 323]

*[My dog is] very funny and I feel like he understands me, he always cheers me up when cuddling me or just generally being around. Also, because since I don’t have siblings it’s like having a cute little brother that’s a lot less annoying that an actual brother would be!* [13yo girl, household 228]

Caregivers and children reflected that their dog had brought their family together through shared physical activity, companionship and laughter. Dog walks were a positive example of this for many families, providing exercise, and an opportunity to spend time together. Caregivers found dog walks useful for extracting children from screens and bedrooms and encouraging conversations that might otherwise have been avoided, and children described enjoying a break from online learning.

*They became playmates and it helped get my son [to] come outdoors for walks and exercise especially during lockdown.* [Female caregiver of one child, household 199]

*A break away from screens. Walks clear the mind. Playing with dogs can be fun.* [15yo girl, household 108]

Some caregivers reflected on aspects of dog ownership they valued. These included the joy they derived from seeing their children happy and their mental health improve, the strengthening of their family bond through sharing the dog, and personal comfort.

*The bond between the dog and my younger child is amazing. He has helped her feel less anxious and she is protective towards the dog. He’s also made us all much happier as a family – taking care of someone together is helpful - he’s like our shared baby.* [Female caregiver of two children, household 91]

*I separated from and divorced the boys’ father during the pandemic. [Dog’s name] has been a fabulous new focus for the boys, who treat her like a furry sibling and adore her. Plus, she’s been a great comfort for me.* [Female caregiver of two children household 374]

#### Theme 2: Owning a dog is harder than we expected….

This theme explores the various challenges described by many caregivers and children of owning a puppy or young dog during, and since, the COVID-19 lockdowns.

***Subtheme 1: Whose responsibility is this?***

Despite many positives, dogs’ care needs exceeded the time and effort many caregivers and some children had anticipated. This appeared particularly evident amongst first-time owners, and during the COVID-19 lockdowns.

*I found the restrictions imposed by having a puppy very difficult. The small amount of time that I had for myself was now for the puppy. I resented this far more than I expected*… [Female caregiver of three children, household 311]

Many caregivers had hoped to delegate at least some their dog’s care to their child(ren); a few felt this was not appropriate. Some hoped that having a puppy would help their child(ren) learn responsibility, though it was not clear whether this had been discussed with or explained to these children. While some caregivers felt their child(ren) had done well, others reflected that they had overestimated how much a relatively young child would want or be able to do with their dog. They also overestimated the degree to which children would be able or willing to follow instructions. Several children described having to take on substantial responsibility for their dog’s care during the COVID-19 lockdowns because their parents were too busy.

*My daughters have learnt responsibility, that his needs always need to be met regardless of anything else and even the less glamorous tasks of picking poo up and rainy walks still have to happen.* [Female caregiver of two children, household 2]

*I had to do a big bit of work because my parents were working full time.* [10yo girl, household 266, reflecting on the COVID-19 lockdowns]

This was not the case in all families. Some children never took the responsibility their caregivers had anticipated; others reduced their involvement post-lockdown. A few caregivers described their child(ren) losing interest in their dog as they entered their teenage years.

*There has been the same level of involvement since the beginning. The relationship is better because the dog has grown up and is better trained so she is more engaged with it, but does not show any level of ownership or responsibility for it. She is busy with her life.* [Female caregiver of one child, household 93]

*I did get the dog for me rather than for the children per se, but I had hoped they would be slightly more involved in his care – perhaps unrealistically! During lockdown, there were fewer distractions, and our activities outside the home were very limited, which probably goes some way to explaining why they were more interested initially.* [Female caregiver of two children, household 264]

In these situations, the predominantly female caregivers reflected on how difficult it had been to add the care needs of a dog to the existing mental load of caring for children, running a household and work. This challenge was particularly evident in reflections on the COVID-19 lockdowns, but several caregivers suggested it was an ongoing issue.

*How much time a dog takes! As in the mental load of having a dog, i.e., vet appts, buying food, worrying about it! Obvs the extra time required for walks. This was especially a problem during the pandemic where I was home schooling 3 children and had to walk the dog. I couldn’t leave them at home but they had lessons and neither did they want to go out in the rain to walk the dog. So it was a constant battle.* [Female caregiver of three children, household 195]

Rarely, children described their dog spending time with others as a downside to ownership. Several caregivers described having to manage a child’s jealousy when a dog appeared to preferentially spend time with adults, when a dog required attention that they wanted, or when a dog appeared to prefer a sibling.

*He resents any attention myself and my husband pay the dog.* [Female caregiver of one child, household 129]

*My dog spends a lot of time with my mum.* [10yo boy, household 329]

Children also identified elements of dog ownership as challenging, particularly puppy behaviours, toilet training, being unable to take the dog into certain public spaces, and dog walks in inclement weather.

*Poo inside on floor standing in it. Crying lots. Mum shouting a lot and smacking him. Keeping me awake.* [8yo boy, household 39]

More surprisingly, children described frustration when their dog sought attention while they were trying to do something independently. Examples included doing homework, watching television, or simply trying to relax. These children effectively characterised unwanted play as a canine problem behaviour for which they did not want to take responsibility.

*I got distracted by [dog’s name] while doing my work for home school.* [8yo girl, household 152]

*She would constantly ask to play when I’m busy.* [12yo boy, household 238]

This did not appear to be something about which caregivers were aware. Whilst several commented on their children finding walks and feeding the dog more work than they anticipated, none mentioned problems their child(ren) experienced with dogs’ attention-seeking behaviour in the house. This contrast is illustrated by the quotes from a caregiver and their child below.

*He has learned a lot by caring for a dog. I don’t know that I can put it into words but he is very proud to be a dog-owner, very interested in dog breeds and [dog’s name] provides a positive constant in his life*. [Female caregiver of one child, household 213]

*I didn’t think owning a dog would be so hard. Owning a dog means I have to spend looking after him and if we didn’t get our dog then I’d have a lot more free time.* [13yo boy, household 213]

***Subtheme 2: Keeping everyone safe***

Many caregivers expressed surprise at how much work it had taken to establish safe boundaries between their new puppy and child(ren). Caregivers, particularly first-time owners, described being taken aback at the frequency and severity of their puppy’s nipping/biting and jumping up behaviours, which they recalled children had found aversive. Unfortunately, several recalled that children’s attempts to run away from their puppy had exacerbated these behaviours. Children also recounted these behaviours when prompted to consider the downsides of having a puppy. Rarely, these behaviours were felt by caregivers to have delayed, or completely precluded, one or more children bonding with their puppy.

*He had really sharp baby teeth which I wasn’t expecting so was a bit scared of him when he jumped up. I know he was only playing but sometimes he would nip and that did hurt. Once his puppy teeth fell out though, he was much better.* [13yo girl, household 185]

*She had loved the school dog but a puppy was a bit too unpredictable for her, and she took a long time to warm up to him. Even now they do not have a good relationship and the dog is, at times, nervous around her*. [Female caregiver of one child, household 295]

Children also needed to be taught how to safely interact with their puppy. Caregivers recalled children ignoring or being unable to interpret canine behaviours indicating their discomfort with an interaction, even those more overt behaviours higher on the ‘ladder of aggression’ such as growling and snapping [50]. Others described children so keen to play that the puppy did not have chance to rest. Some had tried to teach their child(ren) about canine body language, and their puppy’s need their own space. However, enforcing these boundaries was described as difficult or frustrating, particularly with younger children. In this context of dogs clearly not wanting attention, parents did seem to be aware of some risks to both child and dog associated with close physical contact.

*[Our dog] does not like being approached when she is in her bed. I had to teach my daughter not to put her face near the dog as she was smothering her and [Dog’s name] was giving warning growls*. [Female caregiver of one child, household 85]

*My son was at a tricky teenage stage. Combined with strictures of lockdown, he was not very willing to listen to what was needed. If he wanted to cuddle the dog, he did so, even if she was sleeping, despite being asked not to.* [Female caregiver of one child, household 59]

Managing interactions between puppies and visiting children was an additional challenge for some caregivers, particularly poorly socialised 'Pandemic Puppies'. Several caregivers recalled shutting away puppies who reacted to visitors with fear or excessive excitement. Others kept their puppy under close supervision or on the lead around visitors, or distracted them with treats until they had calmed down. Some puppies displayed severe separation-related behaviours, further exacerbating the problem. Many puppies had outgrown these problem behaviours around guests, but some had not.

*He’d still jump up if left alone with visiting children, so I always make sure he’s closely supervised and not able to jump up other children*. [Female caregiver of two children, household 289]

Some families felt their dog’s behaviour consistently disrupted the pleasure they had anticipated would be associated with dog ownership. A few dogs exhibited reactive or other fear-related behaviours in response to other dogs or people at home and/or when out walking, or displayed severe separation-related behaviours. Other dogs were described as pulling so excessively on the lead during walks that caregivers felt it was unsafe for children to hold the lead, let alone walk the dog alone. Children and caregivers described these problem behaviours as creating stress for everyone in the household.

*I couldn’t take him off lead and he would bark at people so walks could be stressful*.[16yo boy, household 235, recalling the COVID-19 lockdowns]

*It was harder to walk [dog’s name] with my child as he wanted to hold the lead but I couldn’t let him as [dog’s name] would have pulled him down and hurt him. So the bonding of the dog and my son ended when we took him out on walks.* [Female caregiver of one child, household 175]

#### Theme 3: One family, one dog, many impacts.

This third theme explores the interconnectedness and dynamism of impacts from dog ownership on families’ mental health. It illustrates the complexity of how a dog’s interactions and relationships with some family members can impact other family members, and how those relationships might evolve with time.

Caregivers alluded to the relationships that they had anticipated different family members having with their dog, and whether those expectations had been met. Several highlighted expected differences between their children, or their dog’s response to different family members.

*[Child 1] isn’t interested in animals and wasn’t interested in this one. [Child 2] loved his last dog and he loves this one even more - it’s been a real success*. [Female caregiver of two children, household 124]

*[Dog’s name]* *has a different relationship with the children typified by trying to chew their slippers (while they’re wearing them) but doesn’t do that with adults*. [Male caregiver of two children, household 226]

Comparing sibling children’s descriptions of their relationships with a single dog also suggests subtly different relationships and interactions. For example, older children were typically more likely to include descriptions of caring and responsibility and younger ones to describe physical interactions and play. This illustrates the different relationships that might simultaneously co-exist between a dog and siblings within a family and might point to how a child’s relationship with their dog could change as they age.

*Something to play with*.[8yo boy, household 286]

*Something to care for, routine in my day.* [13yo girl (sibling of child quoted above), household 286]

The puppies had matured into dogs during the timeframe over which caregivers were asked to reflect. As described in the previous theme, the early puppy stages were challenging for many families, but as the dog aged and their owner-perceived problem behaviours largely resolved, children were typically able to bond with the dogs more closely. This reflects the dog’s age as a factor in changes in child-dog relationships over time.

*At first it was tricky as puppy behaviour was more challenging than we all expected, especially the nipping and chewing. However we got through with lots of consistent training and my daughter now loves our dog and vice versa.* [Female caregiver of one child, household 184]

Whilst typically a dog had been popular with all family members, a few caregivers described specific children or adult partners who were scared, stressed, frustrated or ambivalent about their dog. When a relationship between one household member and a dog was not positive, or if the dog’s primary caregiver was stressed by their care, this could have a collateral impact on other family members. Several female respondents commented on the negative impacts of managing these relationships on their own mental health, and on their ability to spend time on other things.

*[Dog’s name]’s reactivity to dogs and difficulty being left alone has at times severely restricted my life. As a result, I have been less able to attend events at school etc. I have also at times been stressed by the dog and probably less patient with [our] child as a result.* [Female caregiver of one child, household 59]

*The stress the dog has caused. I had assumed she would be a lovely companion for my husband as he works from home but it just caused him more stress which impacted his relationship with the dog. As she is getting older my husband is definitely more settled with her as the dog is more settled, but it has been very hard for me keeping everyone happy!* [Female caregiver of two children, household 38]

Many caregivers summarised complex relationships with their dogs: personal affection for the dog, delight at the relationship their dog had with their children, but also elements of stress and frustration related to aspects their care and/or the dog’s relationships with others.

*I think we felt a bit resentful that the children did not do what they promised to do when they got the dog and help out with him. That probably led to tension as we felt that we’d been hoodwinked a bit and then had a dog that cost us a lot of money and time and we hadn’t been convinced that we wanted him ourselves. But we love him*. [Female caregiver of two children, household 121]

## Discussion

This mixed-methods study provides novel insights into the challenges and benefits for families from sharing their home with a puppy, including through the lens of the COVID-19 ‘Pandemic Puppy’ boom. Caregivers of approximately one quarter of children reported that their child had received a diagnosis of, or support for, a mental health problem – roughly in-line with NHS data which suggests 20.3% of children and young people in the UK had a mental health problem in 2023 [51]. Our qualitative data suggests that parents and children derived joy from dog ownership with dogs described as an indispensable mental health aide by a few parents, and a best friend by many children. However, challenges related to maintaining safe dog-child interactions, mismatched expectations, responsibility for the dog’s care, and the mental load of caring for a puppy plus children were evident.

Most children included in this study described comfort and happiness from living with a dog, results which concur with previous research [10,11,20,52,53]; the importance of dogs as attentive, non-judgemental partners and confidantes for children have been discussed elsewhere [39,54,55]. The necessity, and ability, of children to have close physical contact with their dog was also apparent, reflecting the findings of Baatz *et al*. [38]. That study identified that 7–12-year-old children’s affection and love for their dogs led to cuddles and hugs, though typically during play rather than for emotional comfort as suggested by the current study. Previously suggested motivations for children to cuddle dogs include: children’s perception that dogs “need” to be shown love through hugs, kisses and cuddles, reflecting their own perceived needs [12,56]; that pleasant affective touch with a dog might mechanistically mirroring close physical contact with humans by decreasing cortisol levels and increasing oxytocin levels, [57]; and that children learning how to regulate their emotions may seek social buffering of their stress responses through physical contact with pets [58]. Close physical contact with dogs is therefore likely to be a highly physiologically rewarding, and desirable, interaction for children. This was perhaps heightened further during the COVID-19 pandemic when other social interactions were limited [20]. Caregivers in the current study typically appeared highly supportive of children interacting closely and lovingly with their dogs, perhaps appreciating the dog performing some comforting aspects of childcare on their behalf.

However, dogs can find it stressful to be around children, particularly when children are highly emotional, noisy or seek prolonged close physical contact [38,39,59–61]. Dogs may also experience emotional contagion, reflecting (particularly negative) emotions of those around them [62–64]. Being bitten is therefore a major risk for a child of close physical contact with a stressed or aversive dog [39], and it was concerning that so few of the caregivers in the current study appeared to recognise this. Worryingly, parents interviewed by Baatz *et al*. [38], and caregivers involved in the current study, recalled struggling to stop children having close physical contact with a dog they did recognise to be displaying signs of aversion or distress, including growling. Given that parents [65] and children [60,66,67] may find it challenging to identify behavioural signs of anxiety in their dog, the incidence of interactions between children and dogs displaying aversive behaviours in the current study may have been higher than described. Further studies are needed to better understand the interactions between upset children and their family dogs to determine whether this interaction increases the bite risk, and if so to investigate mitigating strategies. Oxley *et al*. [68] have established that veterinary students can be taught to recognise dog aggression behaviours and change their own behaviour appropriately using a video simulation; similar initiatives may be beneficial for children.

First-time dog owners were revealed to have significantly more unrealistic expectations about the ease of puppy training (particularly their puppy’s nipping and biting), how easy it would be to manage relationships between their puppy and children, and their children’s likely involvement in puppy care. They also comprised 85% of those who had considered relinquishing their dog. Media imagery of the human benefits of dog ownership may lead to unrealistic expectations that puppies and children ‘grow up together’ in families without challenge or effort [69]. Our results suggest that first-time owners may commonly overestimate the ease of integrating a dog into their family home and may require additional support.

In common with previous research [16,20], many caregivers hoped their children would take on some responsibility for their dog’s care, but one in five reported that their child had taken less responsibility than expected, sometimes leaving those caregivers frustrated and stressed. Fifield and Forsyth [16] identified that children in New Zealand whose parents had bought them a dog to teach them about responsibility cared for that dog significantly less often than children with dogs bought for other reasons, though they did not establish why. Children involved in our study suggested that in some instances, expectations were too great. Previous research [12] identified that pet care responsibilities taken by children may be constrained by what parents think is safe or acceptable. Charmaraman *et al*. [10] revealed that in children aged 10–17 years, increased responsibility was significantly associated with a higher likelihood of identifying as a dog’s owner rather than them being a friend or sibling. It might therefore be important for parents to discuss ownership and how responsibility will work with their children prior to acquiring a new puppy, taking into account the age of those children when doing so [10,12,70]. Regular reappraisal may be needed; it was evident in our qualitative data that children’s desire, and perhaps ability, to take responsibility could change substantially over time. Longitudinal studies following the changes in responsibility for a dog’s care within a family over time, and its concurrent impact on their mental health, would be a vital addition to this research field.

Over twenty percent of caregivers had expected their child to play more with their dog. Our qualitative data revealed that multiple children described irritation or frustration at a dog seeking unwanted play with them. Caregivers, on the other hand, all described dog-child play interactions as positive and enjoyable for their children. This suggests these unwanted play interactions may not be observed by caregivers, and/or that children had not discussed these frustrations. Muldoon *et al*. [12] found that some children considered playing with a pet as itself being an aspect of taking responsibility, and their further work [56] identified that children may consider playing with a pet dog as important to that dog’s welfare. Additionally, playing with a dog has been described as a source of positive affect [57], and play was not included in recent lists of problem [71] or negative [72] canine behaviours. However, there is some existing evidence that play may not always be wanted by children; “getting mad with their dog” was logged by children in daily dog-relationship diaries [70]. Children of secondary school age reportedly play with their dogs less than primary school age children [73] so older children may experience more frustration if play-seeking does not diminish accordingly. Our results suggest that a dog seeking play could be a cause of negative affect, particularly in children, if that play is not reciprocally desired, and as such should be added to the list of owner-perceived problem behaviours. Predictors, and consequences, of this should be further explored.

Other owner-perceived canine problem behaviours were recorded, both qualitatively and quantitatively. Challenges posed by puppies jumping up and nipping were particularly prominent. Fortunately, most puppies outgrew these behaviours, but a few caregivers reported permanently damaged relationships between puppies and children because of these behaviours during puppyhood. In addition, several dogs were reactive towards other people or dogs, or pulled excessively on the lead whilst on walks, reducing the mental health benefits of those walks for those families. Unfortunately, unwanted behaviours are linked to relinquishment particularly when those behaviours were not part of owners’ expectations prior to acquiring a dog [74,75]; almost half of the caregivers considering relinquishment in the current study were motivated to do so by their dog’s problem behaviours.

Owners of 96.7% of a cohort of *n* = 985 Pandemic Puppies at the age of 21-months reported one or more persistent owner-reported problem behaviours in those dogs [71]. Causal links were drawn with the unusually isolated start in life of these puppies during the COVID-19 pandemic lockdowns and a concomitant absence of in-person training classes. Given the overlap in populations between that study and the current study, it is likely that some of those dogs were included here. However, our qualitative data shed further light on the negative effects these behaviours had on their families, reflecting the findings of Barcelos *et al*. [2] and Merritt *et al.* [76] that a dog displaying aggressive, anxious or fearful behaviours can be correlated with poorer well-being outcomes for their owners. The importance of dog training classes to reduce the probability of problem behaviours has been identified [71] and should be highlighted to new owners whenever possible. Ideally, they should also include children (either physically, if appropriate, or provide accessible resources for caregivers and children) to increase their awareness of canine body language and safe interactions.

This study offers further proof of concept that inclusion of children in research about the impact of family dogs is possible, and valuable. Extensive research has explored preferences within individual dogs for different people and for other dogs (e.g., [4,77–79]), but only one study [38] has previously sought to understand dog-human relationships beyond the single dog-owner dyad. Our qualitative data found that multiple different person-dog relationships co-exist in the same household, and those relationships change over time as dogs, and children, aged. The impact for adults and children of living with a dog was affected in some instances by other family members’ relationships with that dog and how different individuals adopted dog care responsibility. This research challenges the validity of the standard single dog-owner dyad model of human-dog interaction research. We suggest that future human-dog interaction research be broadened to include everyone living in the same household, and even non co-habiting family members who interact with the dog regularly [56].

In-keeping with a previous report [80], 95% of the dogs’ primary adult caregivers in this study were women, clearly positioning dog care as women’s work. All our female adult respondents were also caring for children. Many women spanned the menopausal age [81], a hormonal change which can be associated with increased feelings of stress and anxiety in addition to disruptive physiological changes. It was evident from the qualitative data that some found the addition of a dog to the household incredibly challenging. In light of growing evidence for a negative impact of the maternal mental load on women’s mental health [82], further work exploring dog ownership through the lens of the maternal mental load, and the menopausal period, is urgently needed, as this may be another risk factor for deteriorating dog-owner relationships, and potentially relinquishment.

Our study design had strengths and limitations. Its major strength was to include the experiences of both caregivers and children within the same household, as only a very small number of previous studies have done so [10,38]. Whilst data in the current study were collected cross-sectionally, respondents were invited to reflect on dog-owner relationships during the COVID-19 lockdowns and at the time of response. In doing so, we were able to capture descriptions of several apparently important drivers of change in dog-child relationships over time.

Our most significant limitation was the number of respondents in the 2019 group which meant we were unable to meaningfully analyse the quantitative mental health and owner-dog bond data collected. We were also unable to find child mental health measures which had been validated for retrospective use, further limiting the reliability of these data, hence their inclusion only in Appendix 2. Families were asked to remember events, relationships and emotions from almost three years prior to the study date, so those data are likely to be subject to recall bias. Additionally, the age limits for children we applied to the study applied at the time of data collection, not during the pandemic; the median age of the respondents who provided free-text data at the time of survey completion was 12 years, meaning these children were nine or ten years old at the start of the COVID-19 pandemic. The impact of the COVID-19 pandemic lockdowns on autobiographical memory, both for events and emotions, is the subject of ongoing research in children and adults [83–87]. This limited evidence to date suggests that most caregivers and children, particularly older ones, would be able to recall events and feelings at those times with some degree of accuracy due to unique nature of national lockdowns. This may have made acquisition of a dog during this period particularly memorable.

We hope to have mitigated some of the recall bias by starting the adult survey with specific questions about household events during the COVID-19 pandemic to provide some anchoring, such as whether individuals were furloughed and whether children were homeschooled. Further bolstering confidence in the validity of our qualitative data, all adult free-text responses contained clear references to the impact of the puppy or dog during the COVID-19 pandemic. Furthermore, almost all responses from children were written in the past tense and contained reference to the dog as a puppy, and/or to events consistent with the COVID-19 pandemic such as the impact of having a puppy when schools were closed, when walks were restricted, or their puppy helping with health anxiety, boredom or mental health problems during the pandemic. This suggests their relationship during the COVID-19 pandemic was being recalled. Even if it were the case that some children described their current relationship with their dog, there is such a dearth of qualitative data capturing child-dog relationships that these responses would still provide valuable insights. A more accurate method of data collection would have been to use contemporaneous daily diaries (e.g., [70]), but the unprecedented nature of the pandemic precluded this degree of academic preparedness.

Children’s qualitative responses were potentially constrained by their language development and writing ability. To mitigate these constraints, adult caregivers were invited to help children with their written responses where needed. We did not capture the total number of children of any age in the household, which would be a useful addition to future surveys. Families may have had additional children who were too old or young to participate but whose relationship with the dog may still have impacted the household, and our data hints that dog-child relationships may be different in single child households. Several parents did include description of dogs’ relationships with all their children, not just those of eligible ages, and this added further useful context. A few families mentioned additional dogs; determining whether the impact of all dogs in a household have an equal impact on families’ mental health would be useful future research.

Our study included elements of selection bias towards dogs that were still owned; the experiences of owners who acquired dogs between 2019 and 2021 then subsequently rehomed or relinquished them may have been substantially different but could not be captured by this study design. As such, more serious, negative impacts that precipitate relinquishment are likely to have been missed. Our sampling strategy incorporated owners of dogs who had previously been involved in our 'Pandemic Puppies' research, along with others recruited online. This self-selecting population could have biased those included towards caregivers of children who were more likely to have mental health problems, those with particularly good or bad memories of the COVID-19 lockdown experiences, and towards those interested in our research. However, our qualitative data suggests we had a wide mix of lockdown experiences, and that the prevalence of children with mental health problems in our population is in line with that of the UK population.

## Conclusions

The COVID-19 pandemic precipitated many families to acquire puppies with the aim of improving their mental health. This study did not find puppy acquisition to be of consistent mental health benefit so this should not be a primary driver of puppy acquisition. Through inclusion of multiple household members in this research, we demonstrated that the consequences to children and caregivers from introducing a puppy to a family home are wide-ranging and may change with time. Individual family members build distinct and unique relationships with the same dog; however, those dog-human relationships, and their impacts on mental health within the family, appear inter-dependent. Rather than exploring single dog-owner dyads, future research on the relationships between dogs and people should include multiple household members to capture this complexity.

Many children benefit positively from their dog’s companionship and affection, though the close physical interactions inherent in many of these dog-child relationships may increase the risk of dog bites. Children may be unprepared for the restrictions and responsibilities associated with dog ownership and may find their dog’s play-seeking behaviours problematic. Caregivers may not anticipate specific challenges associated with having a young dog and children, such as aspects of the dog’s behaviour, setting boundaries for how the children and dog interact, and how responsibility for their care might be negotiated. Specific resources focusing on these potential problems should be developed for families seeking to acquire a new dog, particularly those who are entering dog ownership for the first time. Caring for a dog appears to be primarily women’s work, and the substantial impact of dog ownership on maternal mental health identified here requires further research attention.

## Supporting information

S1 Appendix Survey. (PDF)

S2 Appendix Methods and results relating to the quantitative aspects of the original survey. (DOCX)
