# Supplementary material for: More than just one man and his dog: The many impacts of puppy acquisition on the mental health of families including children in the UK
Source: PLoS One. 2025 Sep 17;20(9):e0331179. doi: 10.1371/journal.pone.0331179 (PMC12443288; doi:10.1371/journal.pone.0331179)
Supplement: S1 Appendix — (PDF) [file pone.0331179.s001.pdf]

S1 File:

Survey

PUBLIC ONLY
COHORT ONLY

Q1 (REQUIRED)
I confirm that:

1. I am over 18 years of age.
2. I am a resident of the UK.
3. I brought a puppy home aged under 16 weeks between 1st January 2019-31st December 2021.
4. I have read and understood the above information and give consent for my answers to be used for this research study and any resulting publications.
5. I have read and understood the above information and give consent for my child's/children's answers to be used for this research study and any resulting publications.

Q2 (REQUIRED)
Do you still own the dog you told us about in the 2020 'Pandemic Puppies' survey (that you brought home between 23rd March-31st December 2020)?
N.B. As a reminder, if you brought home more than one puppy in the time period above, we asked you to answer for the youngest dog, or in the case of littermates, the dog whose name came first alphabetically.

1. Yes – I still have my dog **[SURVEY LOGIC – skip questions 3-15]**
2. No – I have rehomed/sold/given away this dog **[SURVEY LOGIC – skip questions 8-14]**
3. No – my dog has died/been put to sleep **[SURVEY LOGIC – skip questions 3-7 and 13-14]**
4. No – my dog was stolen/lost and is still missing **[SURVEY LOGIC – skip questions 3-12]**

Q3
If you are comfortable doing so, please describe in your own words why you decided to rehome, sell, give away or otherwise stop owning this dog. **[free text]**

Q4
Which month/year did you rehome, sell or give away this dog?
Drop down month list
Drop down year list (2020-2023)

Q5
If you are happy to share with us, please specify how you rehomed, sold or gave away this dog?

1. Rehomed via a multi-breed, multi-location rescue charity (e.g., Dogs Trust, RSPCA, Battersea, Blue Cross, Woodgreen, Jerry Green)
2. Rehomed via a multi-breed rescue organisation not listed above (e.g., local, independent, and/or single-site rescue centres)
3. Rehomed via a breed specific rescue (e.g., English Springer Spaniel Welfare, Border Collie Trust GB, Doodle Trust)
4. Rehomed/given to a family member free of charge
5. Rehomed/given to a friend free of charge
6. Rehomed/given to someone I did not know previously free of charge
7. Privately sold to a new owner via an internet selling site (e.g., Pets4Homes)
8. Privately sold to a new owner I already knew (e.g., friend, family, acquaintance)
9. Other (please specify below) **[free text]**

Q6

Was the rising cost-of-living a factor in this decision?

N.B. If you would like to explain your answer further in your own words, please feel free to do so below. **[free text]**

1. Yes, entirely
2. Yes, partly
3. No, not at all
4. I'm not sure

Q7

Please share any other thoughts or feelings about your experiences of buying and raising a dog during the pandemic and subsequently rehoming, giving away or selling them, including the reasons why, if you feel comfortable to do so. **[free text]**

Q8

We're sorry to hear that your dog is no longer alive. If you feel comfortable doing so, we'd like you to share a bit more about what happened to enable us to help other owners and their dogs. Firstly, how did your dog die?

1. Put to sleep (euthanised) **[SURVEY LOGIC – skip question 10]**
2. Natural or unassisted death (not euthanised/put to sleep) **[SURVEY LOGIC – skip question 9]**
3. Accidental death (e.g., run over) **[SURVEY LOGIC – skip question 9]**
4. Prefer not to say

Q9

If you are comfortable doing so, please describe in your own words what led to the decision for your dog to be put to sleep, including whether the rising cost-of-living was a factor. **[free text]**

Q10

If you are comfortable doing so, please describe in your own words what circumstances led to your dog's death. **[free text]**

Q11

Please indicate which month/year your dog was put to sleep or passed away.

Drop down month list

Drop down year list (2020-2023)

Q12

Are there any other thoughts or feelings about your experiences of buying and raising a dog during the pandemic and subsequently losing them that you are happy to share? **[free text]**

Q13

We're sorry to hear that your dog has been stolen/gone missing. If you feel comfortable doing so, we'd like to ask you to answer a few questions about what happened. Firstly, please indicate which month/year your dog was stolen/went missing.

Drop down month list

Drop down year list (2020-2023)

Q14
Are there any other thoughts or feelings about your experiences of buying and raising a dog during the pandemic and them subsequently being stolen/going missing that you are happy to share? **[free text]**

Q15
Would you like to receive project updates from the RVC about the Pandemic Puppies study?
1. Yes, I would like to receive updates about the research you are carrying out (please do not delete my contact details) **[SURVEY LOGIC – send to the end of the survey]**
2. No, I would rather not hear from you again in the future (please delete my contact details) **[SURVEY LOGIC – send to the end of the survey]**

Q16/Q2 (REQUIRED)
Do you have any children in your household aged 8-17 years inclusive?
1. Yes
2. No **[SURVEY LOGIC – send to the end of the survey]**

Q17 (REQUIRED)
Are you and your child/children willing to take part in this study about your experiences with your dog?
This would greatly help our overall understanding of dog welfare and enable us to help improve the lives of dogs in the future, as well as understanding if and how dogs influence mental wellbeing in children.
N.B. This adult-specific survey will take 10 minutes and an additional 8 minutes per child and is to be completed within 32 days of the initial link being sent via email. You can answer the questions in more than one session.
3. Yes, me and my child/children would like to take part in the current research about my dog and child/children
4. No, but I would like to receive updates and possibly take part in future research you are carrying out (please do not delete my contact details) **[SURVEY LOGIC – send to the end of the survey]**
5. No, I would rather not take part in further research about my dog and would prefer not to hear from you again in the future (please delete my contact details) **[SURVEY LOGIC – send to the end of the survey]**

Q18/Q3
If you are happy to do so, please tell us your dog's name. **[free text]**

Q4 (REQUIRED)
When did you bring your dog home?
(Date text field)
N.B. If you are not entirely sure of the day of the month you brought your dog home, just enter the 1st.

Q5
Please select the breed or crossbreed of your dog from the drop down list.
Drop down list
N.B. If your dog's breed or crossbreed is not included on the list, please describe in the box provided.

Q6
What is your dog's date of birth?
(Date text field)

Q7

What sex is your dog?

Male neutered

Male unneutered (entire)

Female neutered

Female unneutered (entire)

Purchasing your Dog

In this section we would like you to think back to before you purchased your dog and consider these questions about your motivations and expectations at that time.

Q8

What were the main reasons your household wanted to acquire a dog?

Please select **all options** that apply.

1. Companionship for myself
2. Companionship for my children
3. Companionship for other adult(s) in my household
4. Companionship for my other dog(s)
5. To keep me/my family busy
6. To encourage myself/my family to walk and exercise
7. To improve my/my family's mental health
8. Due to the loss of a previous dog in my household
9. As a working dog for a specific role (e.g., gundog, security, sniffer/tracking, herding, medical detection, assistance/therapy dog)
10. For a specific non-working role (e.g., dog sports, showing, etc.)
11. For breeding (including stud dogs)
12. Due to the ageing/ill health of another dog/pet in my household
13. Companionship for other (non-dog) pet(s)
14. Due to the loss of another (non-dog) pet in my household
15. Other reason not listed above (please describe in your own words) **[free text]**

Q9

Did you or your household carry out any research into owning dog and/or which breed/crossbreed to buy before you purchased your puppy?

1. No
2. No - but I was already an experienced dog owner of a different breed/crossbreed
3. No - but I had owned the breed/crossbreed before
4. Yes - please describe in your own words the research you carried out **[free text]**

Q10

<u>What characteristics were you looking for in a dog when selecting a particular breed/crossbreed to buy? Please select **all options** that apply.</u>

1. I'd owned this breed or crossbreed before
2. I grew up with or had childhood experiences with this breed/crossbreed
3. Friends or family currently own this breed/crossbreed
4. Affordable purchase cost of puppies
5. Affordable cost of upkeep
6. Appearance/looks
7. Low grooming needs
8. Low exercise requirements
9. Good with children
10. Good companion
11. Size suited to my lifestyle
12. Generally healthy breed/crossbreed
13. Popularity of the breed/crossbreed
14. Working ability of the breed/crossbreed
15. Long life expectancy
16. Exercise encouragement
17. Celebrity/Influencer endorsement/ownership
18. Hypoallergenic
19. Easy to train
20. I'd always wanted this breed/crossbreed
21. Specific genetic traits/line of the breed/crossbreed
22. Low or non-shedding breed/crossbreed
23. Other temperament/personality traits of the breed/crossbreed
24. None of these options – someone else in the household selected the breed/crossbreed of our dog
25. None of these options – I did not have any specific characteristics I was looking for
26. Other (please tell us here) **[free text]**

Q11

<u>How long after you/your household decided to look for a puppy did you bring your dog home?</u>

1. Less than 1 week
2. Between 1 week-1 month
3. 1 month-6 months
4. >6 months
5. I don't remember

Q12

<u>Who was the driving force in wanting to acquire a puppy?</u>

<u>Please select **all options** that apply</u>

Myself

Another adult in the household

A child or children in the household

All members of the household were equal in their desire to want a puppy

Other (please tell us who here) **[free text]**

Q13

If you brought home your dog after 23rd March 2020, what were the reasons that the COVID-19 pandemic may have influenced you/your households' decision to purchase a puppy?

Please select **all options** that apply

N/A – I purchased my dog before the first COVID-19 lockdown in March 2020

I/we wanted more company due to being at home more

I/we had more time to care for a dog

I/we wanted more company as family and/or friends were unable to visit me/us

I/we wanted a reason to go outside to exercise more

I/we wanted something happy to focus on

I/we were bored due to the restrictions imposed by lockdown

I/we had extra money to spend that I/we would have usually spent on other things

My child/children were at home and I/we wanted something to keep them busy

Other (please tell us why here) **[free text]**

Your Dog's Early Life Experiences

In this section we would like you to think back to soon after you purchased your dog and consider this question about their early life experiences.

Q14

Did you or someone in your household attend any puppy classes with your dog before they were 16 weeks old?

Please select **all options** that apply

1. Yes, in-person puppy classes
2. Yes, online puppy classes
3. No, I wanted to but there weren't any classes running
4. No, I was unable due to my puppy's circumstances (e.g., my puppy was already close to 16 weeks when I brought them home, they hadn't had their vaccinations, they were ill)
5. No, I was unable to attend classes due to my circumstances (e.g., I was ill/isolating, the times were inconvenient)
6. No, I chose not to because I am a dog professional (e.g., trainer, behaviourist)
7. No, other (please describe here)

Children and Dogs Living Together

In this section we would like to understand your experience of getting a dog and having a child/children in your household.

Q19/Q15

Thinking back to when your dog was a puppy, how did the following aspects of their care meet the expectations you had prior to acquiring them? **[free text]**

N.B. If you would like to explain any of your answers further in your own words, you will have the opportunity to do so on the next page.

Rows

a. Toilet training
b. Dealing with biting/chewing of household objects, e.g., furniture, children's toys
c. Dealing with nipping/biting of my child/children during play
d. Managing interactions between my child/children and the puppy

Columns

1. Worse/harder than I expected
2. As I expected
3. Better/easier than I expected
4. I'm not sure/I can't remember

Q20/Q16

Please describe anything else that you found easy, difficult, useful, or surprising about bringing up a dog along with your child/children... **[free text]**

Q21/Q17

Do you have any current concerns about the way your dog and child/children interact?

1. No, and I never have
2. No, not currently, but I have in the past (please describe in your own words below) **[free text]**
3. Yes (please describe in your own words below) **[free text]**

Q22/Q18

Do you have any current concerns about the way your dog and visiting children from outside of your household interact?

1. No, and I never have
2. No, not currently, but I have in the past (please describe in your own words below) **[free text]**
3. Yes (please describe in your own words below) **[free text]**

Q23/Q19

Which of the following is your child/any of your children allowed to do with your dog? Please select **all options** that apply.

Rows

a. Pull on your dog's body parts, e.g., tail, ears
b. Feed your dog their meals
c. Physically correct your dog, e.g., tap your dog's nose, etc.
d. Groom your dog
e. Take away your dog's food (including their bowl and/or dog chews)
f. Hug your dog
g. Throw objects at your dog
h. Stroke or pat your dog on its body
i. Sit, lie or ride on your dog
j. Stroke or pat your dog on its head NOT whilst it is eating or drinking
k. Restrain your dog by its collar (directly, without a lead)
l. Approach or follow your dog
m. Take away your dog's toys
n. Kiss your dog
o. Yell, scream or be noisy whilst playing games with or around your dog
p. Stroke or pat your dog when it is eating or drinking
q. Hold your dog's lead whilst on walks
r. Dress your dog up in human/dolls clothes
s. Lie down near your dog when it is resting
t. Take child toys/possessions away from your dog
u. Give your dog commands (e.g., sit, stay, etc.)
v. Tell your dog off verbally
w. Involve your dog in their own games, e.g., doctors, vets, etc.
x. Wake your dog when it is sleeping
y. Pick your dog up

Columns

1. Always
2. Sometimes
3. Never

Q24/Q20

In hindsight, was/were your child/children the right age for a dog to be introduced to the household?

1. Yes, they were the right age
2. No, I/we should have introduced a dog sooner
3. No, I'd/we'd have rather waited until they were older
4. I'm not sure/I can't remember
5. Other (please describe in your own words below) **[free text]**

Q25/Q21

Have there been any unexpected benefits to owning and raising a dog in a household with children during the pandemic? These can be practical and/or emotional benefits. **[free text]**

Q26/Q22

Have there been any unexpected disadvantages to owning and raising a dog in a household with children during the pandemic? These can be practical and/or emotional challenges. **[free text]**

Q27/Q23

Have you ever seriously considered rehoming your dog?

1. No – I have never considered rehoming them **[SURVEY LOGIC – skip questions 28 and 29]**
2. Yes – but I am no longer considering rehoming them
3. Yes – and I am still considering rehoming my dog

Q28/Q24

If you are comfortable doing so, please describe in your own words why you considered rehoming your dog. **[free text]**

Q29/Q25

If you are comfortable doing so, please describe in your own words what you think would help/have helped you to avoid having to consider rehoming your dog. This could be advice pre- or post-purchase, access to resources, etc. **[free text]**

Your Relationship with Your Dog

In this section we would like to understand **your** current relationship with your dog, **your** current mental health, and **your** mental health during the pandemic.

Q30/Q26

Consider the following statements about your relationship with your dog (as the adult owner), and choose the option that best answers them.

Rows

a. My dog provides me with companionship
b. Having a dog gives me something to care for
c. My dog provides me with pleasurable activity
d. My dog is a source of constancy in my life
e. My dog makes me feel needed
f. My dog makes me feel safe
g. My dog makes me play and laugh
h. Having a dog gives me something to love
i. I get more exercise because of my dog
j. I get comfort from touching my dog
k. I enjoy watching my dog
l. My dog makes me feel loved
m. My dog makes me feel trusted

Columns

1. Strongly agree
2. Agree
3. Disagree
4. Strongly disagree

Q31/Q27

<u>Considering **the last 4 weeks**, choose the option that best matches the statements below.</u>

<u>How often…</u>

Rows

a. Have you felt so down in the dumps that nothing could cheer you up?
b. Have you felt down hearted and blue?
c. Have you been a happy person?
d. Have you been a very nervous person?
e. Have you felt calm and peaceful?

Columns

1. All the time
2. Most of the time
3. A good bit of the time
4. Some of the time
5. A little bit of the time
6. None of the time

Q32/Q28

<u>Thinking back to **the COVID-19 lockdowns**, choose the option that best matches the statements below.</u>

<u>How often…</u>

Rows

a. Did you feel so down in the dumps that nothing could cheer you up?
b. Did you feel down hearted and blue?
c. Were you a happy person?
d. Were you a very nervous person?
e. Did you feel calm and peaceful?

Columns

1. All the time
2. Most of the time
3. A good bit of the time
4. Some of the time
5. A little bit of the time
6. None of the time

You and Your Household

In this section we would like to understand how your living circumstances may have changed since you purchased your dog.

In this section we would like to understand your living circumstances.

We are including some questions about your/your household finances and asking for your postcode (both of which are entirely voluntary).

The reason we are doing this is to assess which dog owners need support, on a regional basis.

Your postcode information will not be analysed at an individual level but across the whole population, you will not therefore be identifiable by giving this information.

Q33

Have you had any changes to the number of dogs in your household since the first survey (in November/December 2020)?

1. No – no changes **[SURVEY LOGIC – skip question 34]**
2. Yes – I now own fewer dogs
3. Yes – I now own more dogs
4. Yes – I now own the same number of dogs, but there has been a change in which dogs I own

Q34

Why has there been a change to the number of dogs in your household since the first survey (in November/December 2020)?

Please select **all options** that apply.

1. A dog(s) in the household died due to old age
2. A dog(s) in the household died for a reason unrelated to old age
3. I sold a dog(s)
4. I rehomed a dog(s) to another household/rescue organisation
5. A dog(s) was stolen/lost
6. I purchased a dog(s)
7. I adopted/rescued a dog(s) into my household

Q35

Has the current composition of your household (e.g., number of adults/children) changed since the first survey (in November/December 2020)?

1. No **[SURVEY LOGIC – skip question 36]**
2. Yes

Q36

How has your household composition changed since the first survey (in November/December 2020)?

1. I now live in a home with both adults and children
2. I now live in a home with children where I am the only adult
3. I now live in a home with more children than before
4. Other (please specify) **[free text]**

Q29

Are you the primary carer for your dog (i.e., the person in your household that provides your dog with the majority of care such as feeding and walking)?

1. Yes
2. No
3. I share the role of primary carer for my dog with someone else in the household
4. I share the role of primary carer for my dog with someone else in a different household

Q30

As an adult, had you ever owned or co-owned a dog before you purchased your dog?

1. Yes
2. No – but someone else in my household had
3. No – I am/everyone in my household was a first-time dog owner(s) when we purchased our dog

Q31

Did you grow up with a dog in your childhood home?

1. Yes
2. No

Q32

How many (if any) other dogs currently live in the same home as your dog?

Please enter a whole number

N.B. If they are the only dog in your household, please type '0'

[number box]

Q33

What best describes your current living situation?

Live in a home with adults and children

Live in a home with children where I am the only adult

Other (please specify) **[free text]**

Q34

How old are you?

1. 18 to 24 years old
2. 25 to 34 years old
3. 35 to 44 years old
4. 45 to 54 years old
5. 55 to 64 years old
6. 65 to 74 years old
7. 75 years old or older

Q35

What is your gender?

1. Female
2. Male
3. Other
4. Prefer not to say

Q37/Q36

What is your highest level of education?

1. GCSEs/O levels
2. AS Levels
3. A-levels
4. Post Graduate Diploma
5. University Degree
6. Masters Degree
7. Doctorate
8. Welsh baccalaureate
9. International baccalaureate
10. Higher grade/advanced higher (Scotland)
11. Certificate of sixth year studies
12. Prefer not to say
13. Other (please specify) **[free text]**

Q38/Q37

What is your overall ANNUAL household income?

1. Up to £10,000
2. £10,001 - £20,000
3. £20,001 - £30,000
4. £30,001 - £40,000
5. £40,001 - £50,000
6. £50,001 - £60,000
7. £60,001 - £70,000
8. £70,001 - £80,000
9. £80,001 - £90,000
10. £90,001 - £100,000
11. More than £100,001
12. Prefer not to say

Q38

Were you/your household affected by the COVID-19 pandemic in any of the following ways?

Please select **all options** that apply

One or more members of my household/I was furloughed

One or more members of my household/I started working from home

One or more members of my household/I became unemployed

Child(ren) had to be home-schooled or cared for from home

None of the above

Prefer not to say

Q39

Were you or any member of your household classed as a key worker during the COVID-19 pandemic?

Please select **all options** that apply

Yes, I was a key worker

Yes, another member of my household was a key worker

No

I prefer not to say

I’m not sure

Q39/Q40

If you are happy to, please provide the first half of your postcode (e.g., AL9 for AL9 7AT) **[free text]**

Q41

We may wish to get in contact with you in the future for limited reasons outlined below.
Please let us know which (if any) you are happy to be contacted about, and provide us with your preferred email address in the box if relevant:

1. To be sent the results of this study
2. To be invited to take part in further research about my dog
3. None of the above

Comment box: [email address]

Thank you for completing this part of the study.

Please press the '**Submit**' button below if you have finished.

Once you click on the '**Submit**' button, the next adult-specific survey will commence, one per child, for **you** to complete.

Once this/these are completed, the child-specific survey for your child/children to complete will begin.

Pandemic Pals – Parent Survey: About Your Child

In this adult-specific survey we will ask you about the child/children in your household aged between 8-17 years inclusive, <u>one at a time</u>.

This survey can be repeated as many times as required if you have multiple children.

QA1 (REQUIRED)
<u>I confirm that:</u>
N.B. If you are a child aged between 8-17 and are reading this, please get your parent/caregiver as this survey is for them to complete. Your child survey will begin after this one is completed.

1. I am over 18 years of age.
2. I am a parent/guardian.
3. If applicable, I understand that I answer this survey for each eligible child in my home ONE AT A TIME.

QA2
<u>Please complete this information for your first/only child.</u>
N.B. This question will be repeated, if applicable for you to tell us about your other children when you repeat the survey.
My child is…

1. 8 years old
2. 9 years old
3. 10 years old
4. 11 years old
5. 12 years old
6. 13 years old
7. 14 years old
8. 15 years old
9. 16 years old
10. 17 years old

QA3
My child is…

1. Male
2. Female
3. Other
4. Prefer not to say

QA4
<u>Is this child from a multiple birth (e.g., twin, triplet)?</u>

1. No
2. Yes – please describe below

Thinking About Your Child…

We are interested in how dogs' impact upon the mental wellbeing of the children they live with. As part of this, we would like to explore your child's mental wellbeing both now (in the past week), and during the lockdown phases of the COVID-19 pandemic. The questions below capture this using established questionnaire tools developed by child psychologists.

Please answer the following questions to the best of your knowledge, ensuring that the answers you give reflect the perspective of your child.

Firstly, please try to remember your child's experiences over the last week...

QA5

Thinking about the last week for your child…

Rows

a. Has your child felt fit and well?
b. Has your child got on well at school?

Columns

1. Not at all
2. Slightly
3. Moderately
4. Very
5. Extremely

QA6

Thinking about the last week for your child…

Rows

a. Has your child felt full of energy?
b. Has your child felt sad?
c. Has your child felt lonely?
d. Has your child had enough time for themselves?
e. Has your child been able to do the things that they want to do in their free time?
f. Has your child felt that their parent(s)/guardian(s) treated them fairly?
g. Has your child had fun with their friends?
h. Has your child been able to pay attention?

Columns

1. Never
2. Seldom
3. Quite often
4. Very often
5. Always

QA7

In general, how would your child currently rate their health?

1. Excellent
2. Very good
3. Good
4. Fair
5. Poor

Now, thinking back to the lockdown phase of the COVID-19 pandemic, please think about these questions again for your child.

QA8

Thinking about the first COVID-19 lockdown in March-June 2020 for your child…

Rows

a. Did your child feel fit and well?
b. Did your child get on well at school? (N.B. this includes periods of virtual/home-schooling).

Columns

1. Not at all
2. Slightly
3. Moderately
4. Very
5. Extremely

QA9

Thinking about the first COVID-19 lockdown in March-June 2020 for your child…

Rows

a. Was your child full of energy?
b. Was your child sad?
c. Was your child lonely?
d. Did your child have enough time for themselves?
e. Was your child able to do the things that they wanted to do in their free time?
f. Did your child feel that their parent(s)/guardian(s) treated them fairly?
g. Did your child have fun with their friends? (N.B. this includes online gaming, social media, etc.).
h. Was your child able to pay attention?

Columns

1. Never
2. Seldom
3. Quite often
4. Very often
5. Always

QA10

In general, how would your child have rated their health during the first COVID-19 lockdown in March-June 2020?

1. Excellent
2. Very good
3. Good
4. Fair
5. Poor

QA11

If there is anything else you would like to tell us about your child's mental and physical health during the first COVID-19 lockdown in March-June 2020, please feel free to do so. **[free text]**

QA12

Have you ever sought support or a diagnosis for your child's mental health from NHS services (CAHMS, etc.) or privately?

1. No **[SURVEY LOGIC – skip question A13]**
2. Yes

QA13

Do you think the COVID-19 pandemic had any effect on your child needing mental health support?

1. No – not at all
2. Yes – a little bit
3. Yes – quite a bit
4. Yes – very much
5. Yes – it was the main reason

Your Child's Relationship with Your Dog

We would like to understand how getting a dog may or may not have helped your child's mental wellbeing.

QA14

Was your dog bought to help improve your child's mental wellbeing?

1. Yes – mainly
2. Yes – in part
3. No – not specifically **[SURVEY LOGIC – skip question A15]**
4. No – not at all **[SURVEY LOGIC – skip question A15]**

QA15

Before you purchased your dog, in what ways did you think owning a dog would improve your child's mental wellbeing?

Please select **all options** that apply.

1. Give my child something to focus upon
2. Encourage my child to do exercise
3. Encourage my child to get out of the house
4. Give my child something to take responsibility for
5. Give my child companionship
6. Other (please describe below) **[free text]**

QA16

Has having your dog changed your child's mental wellbeing?

1. Yes – it has made it a lot better **[SURVEY LOGIC – skip question A17**
2. Yes – it has made it a little bit better **[SURVEY LOGIC – skip question A17**
3. No – it has not really made any difference **[SURVEY LOGIC – skip question A17 and A18]**
4. Yes – it has made it a little bit worse **[SURVEY LOGIC – skip question A18]**
5. Yes – it has made it a lot worse **[SURVEY LOGIC – skip question A18]**
6. I'm not sure **[SURVEY LOGIC – skip question A17 and A18]**

QA17

Please describe in your own words how your dog has made your child's mental wellbeing worse.

[free text]

QA18
Please describe in your own words how your dog has improved your child's mental wellbeing. **[free text]**

Your Child's Involvement with Your Dog

We would like to understand how getting a dog and managing them with your child may or may not have met your expectations.

QA19
For this question we would like you to think back to when you first brought home your dog, and about how much your child was involved in the following activities with them until your dog was 6 months old.

Compared to my expectations, the amount my child was involved when we first acquired our dog in...
Rows
a. Feeding them
b. Walking them
c. Grooming them
d. Taking responsibility for them
e. Playing with them

Columns
1. Was less than I expected
2. Was as I expected
3. Was more than I expected
4. I'm not sure/I can't remember

QA20
If you would like to further explain any of your answers to the above question, please feel free to do so. **[free text]**

QA21
For this question we would like you to answer about the amount your child is involved in the following activities now.

Compared to my expectations when we first acquired our dog, the amount my child is **now** involved in...
Columns
a. Is less than I expected
b. Is as I expected
c. Is more than I expected
d. I'm not sure/I can't remember

Rows
1. Feeding them
2. Walking them
3. Grooming them
4. Taking responsibility for them
5. Playing with them

QA22

If you would like to further explain any of your answers to the above question, please feel free to do so. **[free text]**

QA23

Compared to when we first acquired my dog, the relationship my child has had with our dog has been... **[free text]**

N.B. If you would like to explain your answer further in your own words, please feel free to do so.

1. Worse than I expected
2. As I expected
3. Better than I expected
4. I'm not sure/I can't remember

Thank you for completing this part of the study.

If you have more eligible children in your household, you can select the 'Take this survey again to tell us about another child in your household.' button below to go on and complete this survey for each of them.

If not, the survey(s) for your child/children to complete will begin next (child-specific survey), once you have selected the '**Submit**' button below.

If relevant, the child-specific survey can be repeated as many times as necessary to allow all eligible children in your household to take part.

QC1

We need to make sure you understand and are happy to take part in this study. Please tick the boxes next to the statements below if you agree with them. If you have any more questions about what we are doing, please ask your parent/caregiver. If they don't know the answer, they can contact us to find out for you.

4. I am happy to take part in this survey.
5. I understand what this survey is about.
6. I understand that my answers will be kept private.
7. I understand that the scientists will write about this research to share the results with others.

About Me and My Dog

We would like to hear all about the time you spend with your dog. Remember there are no right or wrong answers. The questions are all about the dog that joined your family in 2020 and you can check which dog this is, if you have more than one, with your parent/caregiver.

QC2

How old are you?

1. 8 years old
2. 9 years old
3. 10 years old
4. 11 years old
5. 12 years old
6. 13 years old
7. 14 years old
8. 15 years old
9. 16 years old
10. 17 years old

QC3

Are you a boy or a girl?

1. Girl
2. Boy
3. Other
4. Prefer not to say

QC4

Think about the activities below and chose which option best matches how often you have done them in the last month.

How often have you…

Rows

a. Walked your dog (on your own or with other family members/friends).
b. Brushed your dog?
c. Fed your dog?
d. Picked up your dog's poo?

Columns

1. Twice a day
2. Once a day
3. A few times a week
4. Once a week
5. Less than once a week
6. Never

QC5

On an average weekday (Monday-Friday), how long do **you** spend playing, walking or just being close to/near your dog?

1. No time
2. 1-15 minutes
3. 16-30 minutes
4. 31-60 minutes
5. 1 or 2 hours
6. 3 or 4 hours
7. 5 hours or more

QC6

On an average weekend day (Saturday-Sunday), how long do **you** spend playing, walking or just being close to/near your dog?

1. No time
2. 1-15 minutes
3. 16-30 minutes
4. 31-60 minutes
5. 1 or 2 hours
6. 3 or 4 hours
7. 5 hours or more

QC7

We would like you to read and think about the 9 sentences below. For each one, let us know how much you agree or disagree with them. **[SAPS Q's]**

Rows

a. I don't really like animals
b. I spend time every day playing with my dog
c. I sometimes talk to my dog and understood what it was trying to tell me
d. I love dogs
e. I talk to my dog quite a lot
f. My dog makes me feel happy
g. I consider my dog to be a friend
h. My dog knows when I'm upset and tries to comfort me
i. There are times I'd be lonely without my dog

Columns

1. Strongly agree
2. Agree
3. Not sure
4. Disagree
5. Strongly disagree

About My Feelings

We would like to find out about your current feelings during the last week and also how you felt during the COVID-19 pandemic, which we know was a difficult time for many children.
Please read every question carefully. What answer comes to your mind first? Choose the answer that fits your answer best.

Remember: This is not a test so there are no wrong answers.

You do not have to show your answers to anybody if you don't want to. Also, nobody who knows you will look at your survey once you have finished it and nobody will be able to identify you from your answers.

QC8

Thinking about the last week…**[KIDSCREEN-10]**

Rows

a. Have you felt fit and well?
b. Have you got on well at school?

Columns

1. Not at all
2. Slightly
3. Moderately
4. Very
5. Extremely

QC9

<u>Thinking about the last week…**[KIDSCREEN-10]**</u>

Rows

a. Have you felt full of energy?
b. Have you felt sad?
c. Have you felt lonely?
d. Have you had enough time for yourself?
e. Have you been able to do the things that you want to do in your free time?
f. Have your parent(s)/caregiver(s) treated you fairly?
g. Have you had fun with your friends?
h. Have you been able to pay attention?

Columns

1. Never
2. Seldom
3. Quite often
4. Very often
5. Always

QC10

<u>In general, how would you say your health is? **[KIDSCREEN-10]**</u>

1. Excellent
2. Very good
3. Good
4. Fair
5. Poor

QC11

<u>Thinking back to the COVID-19 pandemic lockdowns, when school was closed for most children…**[adapted from KIDSCREEN-10]**</u>

Rows

a. Did you feel fit and well?
b. Did you got on well at school, including your online lessons?

Columns

1. Not at all
2. Slightly
3. Moderately
4. Very
5. Extremely

QC12

Thinking back to the COVID-19 pandemic lockdowns, when school was closed for most children…**[adapted from KIDSCREEN-10]**

Rows

a. Did you feel full of energy?
b. Did you feel sad?
c. Did you feel lonely?
d. Did you have enough time for yourself?
e. Were you able to do the things that you wanted to do in your free time?
f. Did you feel your parent(s)/caregiver(s) treated you fairly?
g. Did you have fun with your friends, including online?
h. Were you able to pay attention?

Columns

1. Never
2. Seldom
3. Quite often
4. Very often
5. Always

QC13

Thinking back to the COVID-19 pandemic lockdowns, when school was closed for most children in general, how would you say your health was? **[adapted from KIDSCREEN-10]**

1. Excellent
2. Very good
3. Good
4. Fair
5. Poor

QC14

During the COVID-19 pandemic lockdowns, when school was closed for most children, did your dog make your life…

1. A lot better
2. Better
3. No different
4. Worse
5. A lot worse
6. I'm not sure

QC15

Think of all the reasons that having a dog made things better during the COVID-19 pandemic lockdowns and type them answer in the box. **[Free Text]**

QC16

Think of all the reasons that having a dog made things worse during the COVID-19 pandemic lockdowns and type them answer in the box. **[Free Text]**

Thank you so much for taking part in our survey. You have helped us in a real scientific study, and we are very grateful to you.

The survey has now ended so you can let your parent/caregiver know if they are not with you at the moment.

Dear **parent/caregiver, if you have more eligible children in your household who would like to take part, please select the 'Please select this button to repeat the survey for other eligible children in your household.' button below.**

If you do not have any more eligible children in your household, please select the 'Submit' button to finish the survey.
