# Supplementary material for: More than just one man and his dog: The many impacts of puppy acquisition on the mental health of families including children in the UK
Source: PLoS One. 2025 Sep 17;20(9):e0331179. doi: 10.1371/journal.pone.0331179 (PMC12443288; doi:10.1371/journal.pone.0331179)
Supplement: S2 Appendix — (DOCX) [file pone.0331179.s002.docx]

**Appendix S2: Methods and results related to the quantitative measures of mental health included in the survey which have been removed from the main text due to being underpowered.**

**Methods**

The surveys were designed in three parts as detailed below. In summary, the first two parts were designed to be completed by the caregiver, focussed on 1) their own relationship with the dog and their own mental health; 2) their child/children‘s relationship with the dog and mental health; and the third part, based on the child’s self-reflection of mental health and relationship with the dog, to be answered by any or all of their eligible children.

Caregiver questionnaire 1 contained the following quantitative measures:

*(i*) *Caregiver-dog bond*: The Comfort from Companion Animals Scale (CCAS) [49] measured the current closeness/intimacy dimension of the human-animal bond at the time of survey completion. CCAS comprises 11 statements such as ‘My pet provides me with companionship’. Agreement with statements is scored on a four-point Likert scale from 1 = strongly disagree, to 4 = strongly agree. The total score range is 11 – 44 with higher scores indicating a stronger bond. CCAS has been externally validated in different population groups with Cronbach’s alpha values over 0.90 [50]. CCAS has been demonstrated to correlate with other established measures of human-animal relationships such as the Lexington Attachment to Pets Scale [49]. Cronbach’s alpha coefficient of CCAS in the current study was 0.92 indicating high internal consistency (reliability) of these scores.

(*ii*) *Caregiver mental health*: The Mental Health Inventory (MHI-5 [51]) was completed by caregivers twice, first considering their mental health in the four preceding weeks, then recalling how they had felt during the COVID-19 lockdowns (questions all beginning “Thinking back to the COVID-19 lockdowns, how often…” as described previously [52]). MHI-5 encompasses five questions all beginning “How much of the time in the previous four weeks have you…” with agreement with statements scored on a six-point Likert scale. Three questions (“…been a very nervous person?”; “…felt so down in the dumps that nothing could cheer you up?” and “…felt downhearted and blue”) are scored from 1 = none of the time, to 6 = all of the time and two (“…felt calm and happy?” and “…been a happy person?”) are scored from 6 = none of the time, to 1 = all of the time. The total score range is 5 – 30 with higher scores representing better mental health. MHI-5 has been used extensively in the UK with Cronbach’s alpha values ranging from 0.74 – 0.88 [53]. MHI-5 has been validated in UK populations [51] and used retrospectively [52]. Cronbach’s alpha coefficient of MHI-5 for the preceding four weeks in the current study was 0.83 indicating high internal consistency (reliability) of these scores. However, Cronbach’s alpha coefficient of MHI-5 when recalling lockdown in the current study was 0.43 suggesting it is less reliable retrospectively despite its previous use in this manner. Results should therefore be interpreted accordingly.

Caregiver Questionnaire 2 additionally included the following:

1. *Child mental health:* For each eligible child, caregivers completed the adult portion of KIDSCREEN-10 (proxy K-10) [54] which provides a global one-dimensional score (range 10 – 50), where a higher score is indicative of a better quality of life. Proxy K-10 comprises ten items about that child’s health such as ‘Thinking about the last week, has your child felt fit and well?’ Responses are scored on a Likert scale with eight questions scored from 1 = not at all/never, to 5 = extremely/always and two questions (“…has your child felt sad?” and “…has your child felt lonely?) scored from 1 = always to 5 = never. Proxy K-10 is a widely used tool, validated in 13 European countries including the UK with a Cronbach’s alpha of 0.78 [55]. In the current study, caregivers reported each child’s wellbeing both at the time of the survey, and recalling how the child felt during the first UK national COVID-19 lockdown (2020). To the authors’ knowledge, proxy K-10 has not previously been used retrospectively and results should be interpreted with caution. In the current study, Cronbach’s alpha coefficient of proxy K-10 at the time of the survey (in 2023) was 0.84, and 0.83 when recalling the first UK national COVID-19 lockdown in 2020, indicating high internal consistency (reliability) of these scores. Finally in this section, a series of multiple choice and free-text questions captured caregivers’ pre-purchase beliefs about how a dog would impact that child's mental health, and their reflections on the dog's impact on that child's mental wellbeing at the time of the survey.

Child Questionnaire: This questionnaire was completed by each eligible child who agreed to participate once their caregiver had completed their questionnaires. The children completed:

*Child mental health*: The KIDSCREEN-10 (self-report K-10) [54] assessed subjective health and well-being. It comprises ten items about the health of a child aged 8-18 years such as “Thinking about the last week, have you felt fit and well?” Responses are scored on a Likert scale with eight questions scored from 1 = not at all/never, to 5 = extremely/always and two questions (“…have you felt sad?” and “…have you felt lonely?”) scored from 1 = always to 5 = never. The self-report K-10 provides a global one-dimensional score (range 10 – 50), where a higher score is indicative of a better quality of life. In our study, children reported wellbeing at the time of the survey and recalling how they felt during the COVID-19 lockdowns; this was framed as “Thinking back to the COVID-19 pandemic lockdowns, when school was closed for most children”. Self-report K-10 validation and prior use is as per proxy K-10, with a Cronbach’s alpha of 0.82 [55]. Intraclass correlation coefficient (ICC) of the proxy and self-report K-10 in this study were 0.63 (in 2023) and 0.55 (lockdown) indicating moderate agreement, as per previous studies [55]. KIDSCREEN has not previously been used retrospectively so results should be interpreted with caution.

1. *Child-dog bond*: The ‘Short Attachment to Pets Scale’ (SAPS) [56,57] explored the strength of children’s attachment to their dog at the time of survey completion. SAPS consists of nine items covering: friendship/companionship; mutual understanding; shared activities; and emotional support. Responses are on a 5-point Likert scale ranging 1 = strongly agree to 5 = strongly disagree; a mean score is calculated across the nine items. SAPS was developed in a UK population of children, with a Cronbach’s alpha of 0.89 [56] with a subsequent study investigating its reliability and validity in relation to the Health Behaviour in School-Aged Children Survey obtaining a Cronbach’s alpha value of 0.85 [58]. Cronbach’s alpha coefficient of SAPS in the current study was 0.82 indicating high internal consistency (reliability) of these scores.

*Quantitative data analysis*

Raw quantitative data were exported from REDCap into Microsoft Excel for Mac, v16.86 for manual cleaning prior to analysis. Responses from ineligible respondents and those whose survey was blank after the consent questions were removed. If respondents had not completed in full any of the question sets relating to permitted child-dog activities and interactions, expectations versus realities of puppy ownership, CCAS, SAPS, MHI-5, or proxy K-10 their responses were excluded from the analysis of that incomplete scale. Incomplete responses were otherwise included, including eligible caregiver responses with no corresponding child responses. IBM SPSS Statistics v29 (SPSS Inc., Chicago, IL., USA) was used to calculate descriptive statistics. Data distribution was assessed visually using histograms. Non-normally distributed continuous data (child age, CCAS, SAPS) are reported as median (IQR, range), while normally distributed data (proxy and self-report K-10, MHI-5) are reported as mean (SD).

For univariable analysis chi-squared (*X^2^*) tests were used for categorical variables whilst Mann-Whitney U tests were applied for binary variables with non-normally distributed continuous variables (CCAS, SAPS), and independent samples t-tests for normally distributed continuous variables (proxy and self-report K-10, MHI-5). Pearson correlation coefficient was used for continuous variable correlations (MHI-5 at both time points, proxy and self-report K-10 at both timepoints, CCAS and MHI-5 and SAPS). Spearman rank order correlation (rho) was used to explore the relationship between child age and SAPS due to non-normal distribution. Chi-squared analysis of caregiver expectations versus realities of puppy training/management and child involvement in dog care at the time of the survey by dog ownership experience included Bonferroni-corrections and post-hoc comparisons. Statistical significance for all quantitative analyses was set at *p* < 0.05.

Table S1. Descriptive and univariable analysis of caregiver MHI-5 at both time points; proxy K-10 at both time points; caregiver CCAS and child SAPS by acquisition group. Significant differences at the 5% level are highlighted by emboldened text

| **Total MHI-5 score at the time of survey**  (2019: *n* = 53; Pandemic Puppy: *n* = 281) | **/** | **Mean = 22.30**  **SD = 4.18** | **Mean = 23.57**  **SD = 3.65** | ***t* = -2.258** | **0.025** |
| --- | --- | --- | --- | --- | --- |
| **Total MHI-5 score when recalling COVID-19 lockdowns**  (2019: *n* = 52; Pandemic Puppy: *n* = 283) | / | Mean = 21.69  SD = 3.87 | Mean = 21.66  SD = 4.38 | *t* = 0.049 | 0.961 |
| **Total proxy K-10 score at the time of survey**  (2019: *n* = 49; Pandemic Puppy: *n* = 286) | / | Mean = 38.41  SD = 6.08 | Mean = 39.24  SD = 5.40 | *t* = -0.979 | 0.328 |
| **Total proxy K-10 score when recalling COVID-19 lockdowns**  (2019: *n* = 47; Pandemic Puppy: *n* = 285) | / | Mean = 36.28  SD = 5.92 | Mean = 35.68  SD = 6.18 | *t* = 0.612 | 0.541 |
| **Total self-report K-10 score at the time of survey**  (2019: *n* = 32; Pandemic Puppy: *n* = 164) | / | Mean = 38.06  SD = 6.29 | Mean = 38.79  SD = 5.93 | *t* = -0.625 | 0.532 |
| **Total self-report K-10 score when recalling COVID-19 lockdowns**  (2019: *n* = 33; Pandemic Puppy: *n* = 167) | / | Mean = 36.06  SD = 6.91 | Mean = 35.87  SD = 6.33 | *t* = 0.152 | 0.879 |
| **Total CCAS score at the time of survey**  (2019: *n* = 51; Pandemic Puppy: *n* = 227) | / | Median = 41.00  IQR = 36.00 – 44.00 | Median = 42.00  IQR = 37.00 – 44.00 | U = 6527.500 | 0.189 |
| **Mean SAPS score at the time of survey**  (2019: *n* = 33; Pandemic Puppy: *n* = 174) | / | Median = 1.89  IQR = 1.67 – 2.25 | Median = 1.94  IQR = 1.67 – 2.33 | U = 2889.000 | 0.954 |

*Quantitative assessment of caregiver mental health*

Pandemic Puppy and 2019 group caregivers reported no significant difference in MHI-5 score when recalling the COVID-19 lockdowns, but Pandemic Puppy owners reported a significantly higher MHI-5 score (i.e., better mental health) at the time of survey completion (post-pandemic) than caregivers in the 2019 puppy group (Table S1). MHI-5 scores from the COVID-19 lockdowns and from the time of survey completion were significantly positively correlated (Pearson’s: 0.56, *n* = 326, *p* < 0.001).

*Quantitative assessment of child mental health*

No significant differences in proxy or self-report K-10 scores were detected between the two acquisition groups either when recalling the first UK national COVID-19 lockdown (2020) or at the time of survey completion (Table S1). Proxy K-10 and self-report K-10 scores at the two timepoints were significantly correlated (proxy scores: Pearson’s: 0.62, *n* = 324, *p* < 0.001; self-report scores: Pearson’s: 0.54, *n=* 192, *p* < 0.001).

The proxy K-10 score of children whose caregivers reported that they acquired their puppy to improve their family’s mental health (54.6%, *n* = 183/335) did not differ from those whose caregiver did not acquire their puppy influenced by this motivation (*n* = 152) both at the time of survey completion (proxy K-10-2023 mean (SD); mental health acquisition: 38.83 (5.85) versus non-mental health acquisition: 39.47 (5.05); *t* = 1.09, df = 333, *p* = 0.277), and during lockdowns (proxy K-10-lockdown; mental health acquisition: 35.29 (6.45) versus non-mental health acquisition: 36.35 (5.71), *t* = 1.58, df = 330, *p* = 0.136).

However, the self-report K-10 score of children whose caregivers reported that they acquired their puppy to improve their family’s mental health (27.0%, *n* = 54/200) was significantly lower than those whose caregivers did not acquire their puppy influenced by this motivation (*n* = 146) both at the time of survey completion (2023) (self-report K-10-2023 mean (SD); mental health acquisition: 36.98 (6.41) versus non-mental health acquisition: 39.25 (5.74); *t* = 2.34, df = 194, *p* = 0.020), and during lockdowns (self-report K-10-lockdown; mental health acquisition: 34.37 (5.00) versus non-mental health acquisition: 36.47 (6.50), *t* = 2.08, df = 198, *p* = 0.039).

*The relationship between mental health and human-dog bond*

The mean CCAS scores for caregivers and SAPS scores for children suggested both had strong bonds with their dogs. The CCAS scores for caregivers in 2019 and Pandemic Puppy groups at the time of survey completion did not significantly differ (Table S1). CCAS was not significantly correlated with MHI-5 score when recalling the COVID-19 lockdowns (Pearson’s: -0.10, *p* = 0.072) or at the time of survey completion (Pearson’s: -0.06, *p* = 0.288).

There was no significant difference in mean SAPS scores at the time of survey completion between children in the two acquisition groups (Table S1). The SAPS of children whose caregivers reported acquiring their dog to provide mental health support for their family (26.6%, *n* = 207) did not differ from those whose caregiver did not acquire their dog influenced by this motivation (median SAPS score; family mental health motivation: 1.89 (IQR: 1.67 – 2.33) versus non-family mental health motivation: 2.00 (IQR: 1.67 – 2.33); U = 3964.5, *p* = 0.570)).

SAPS was not significantly correlated with proxy or self-report K-10 scores when recalling the COVID-19 lockdowns (proxy: Pearson’s: -0.05, *n* = 200, *p* = 0.511; self-report: Pearson’s: 0.01, *n* = 197, *p* = 0.988 ) or at the time of survey completion (proxy: Pearson’s: -0.04, *n* = 198, *p* = 0.577; self-report: Pearson’s: -1.31, *n* = 194, *p* = 0.068). SAPS was also not significantly correlated with child age (*rho* = 0.11, *n* = 205, *p* = 0.124) and was not detected to significantly differ between male and female children (U = 5834.0, *n* = 206, *p* = 0.079).
